# *TP53BP1,* a dual-coding gene, uses promoter switching and translational reinitiation to express a smORF protein

**DOI:** 10.1016/j.isci.2023.106757

**Published:** 2023-04-27

**Authors:** Marta A. Inchingolo, Aurélie Diman, Maxime Adamczewski, Tom Humphreys, Pascale Jaquier-Gubler, Joseph A. Curran

**Affiliations:** 1 Department of Microbiology and Molecular Medicine, Faculty of Medicine, University of Geneva, Geneva, Switzerland; 2 Faculté de Médecine et Pharmacie, Université Grenoble Alpes, Grenoble, France; 3 Faculty of Biology, Medicine and Health, University of Manchester, Manchester, UK; 4 Institute of Genetics and Genomics of Geneva (iGE3), University of Geneva, Geneva, Switzerland

**Keywords:** Protein, Molecular biology, Cell biology

## Abstract

The complexity of the metazoan proteome is significantly increased by the expression of small proteins (<100 aa) derived from smORFs within lncRNAs, uORFs, 3′ UTRs and, reading frames overlapping the CDS. These smORF encoded proteins (SEPs) have diverse roles, ranging from the regulation of cellular physiological to essential developmental functions. We report the characterization of a new member of this protein family, SEP53BP1, derived from a small internal ORF that overlaps the CDS encoding 53BP1. Its expression is coupled to the utilization of an alternative, cell-type specific promoter coupled to translational reinitiation events mediated by a uORF in the alternative 5′ TL of the mRNA. This uORF-mediated reinitiation at an internal ORF is also observed in zebrafish. Interactome studies indicate that the human SEP53BP1 associates with components of the protein turnover pathway including the proteasome, and the TRiC/CCT chaperonin complex, suggesting that it may play a role in cellular proteostasis.

## Highlights

• *TP53BP1* mRNA transcripts from an alternative promoter carry a uORF within the 5′ TL
• The uORF promotes delayed reinitiation events at an internal overlapping ORF (ioORF)
• The ioORF expresses a 50 aa Small ORF Encoded Protein (SEP53BP1)
• The SEP53BP1 interactome includes the proteasome and the TRiC/CCT chaperonin

## Graphical abstract

**Figure:**
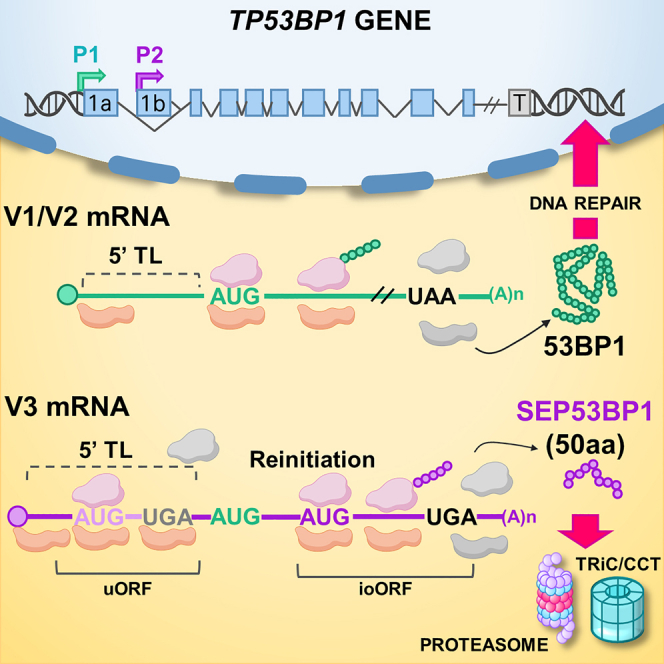

## Introduction

Protein synthesis represents a key step in the regulation of gene expression. The differential recruitment of mRNA populations onto polysomes permits a rapid response to changes in the cellular environment. As such, it is a key process in the maintenance of homeostasis, and perturbations in its control are associated with numerous disorders. Translation can be subdivided into four main steps: initiation, elongation, termination and subunit recycling. Most regulation is exerted at initiation, and this has been confirmed in translational profiling studies covering the entire mammalian transcriptome.^1^ The ternary complex (TC) composed of Met-tRNAi-eIF2-GTP is first loaded onto the 40S ribosomal subunit in combination with a series of eukaryotic initiation factors (eIFs) to form the 43S pre-initiation complex (PIC). The PIC is generally loaded onto the mRNA via the 5′ cap. Once recruited, it moves forward (5′→3′) scanning the mRNA 5′ TL or UTR (Transcript Leader or UnTranslated Region) to locate the first AUG. The nucleotides flanking the AUG codon influence the efficiency of recognition, with the sequence 5′-ACCAUGG-3′ (the Kozak context: the nt in red being particularly important) being optimal in mammals.^2^ If sub-optimal, scanning ribosomes will sometimes ignore the AUG codon and continue to the next. This phenomenon, known as leaky scanning, can produce N-terminal truncated proteins or proteins from overlapping reading frames.^3^^,^^4^

The 5′ TL contains a number of features that can regulate the translational readout during both PIC recruitment and subsequent scanning.^5^ This includes uAUGs and uORFs (upstream Open Reading Frames). Genomic analysis has estimated that ∼50% of human 5′ TLs contain one or more uORFs.^6^^,^^7^ Both uAUGs and uORFs can function as translational repressors limiting PIC access to downstream start codons.^8^ The amplitude of this repression is dictated by the uAUG context.^9^ However, small uORFs (<50 codons) can also couple the readout to stress and TC levels in the cell, via a process referred to as delayed reinitiation in which the 40S ribosome remains on the mRNA and continues to scan subsequent to translation of the uORF. This process permits access to start codons downstream of the AUG of the principle CDS (AUG-CDS).^7^^,^^10^ However, the efficiency of reinitiation at downstream start sites varies depending on parameters such as uORF length and the distance between the stop codon and the AUG. This process is conserved from human to zebrafish.^11^ Reacquisition of the Met-tRNA by the 40S ribosome after uORF termination is dependent on eIF2-GTP levels. When low, the slow reacquisition can cause a bypass of a proximal downstream AUG as the 40S is unable to re-recruit the TC necessary to form an initiation competent PIC. The TC levels respond to stress via the regulation of a series of “stress activated protein kinases” that include GCN2, HRI, PKR and PERK. These form the axe of the “integrated stress response” (ISR). Their substrate is the α subunit within eIF2.GDP generated during each round of translational initiation.^12^^,^^13^ Phosphorylation impedes GDP/GTP exchange and the subsequent TC regeneration. Thus, reinitiation in combination with leaky scanning offers the possibility to significantly increase the complexity of the mammalian proteome by permitting access to internal AUGs (iAUG).

Alterations in the 5′ TL arise because of the use of alternative promoters (AP), transcriptional start site (TSS) heterogeneity and alternative splicing,^14^^,^^15^^,^^16^ with studies suggesting that AP exceeds alternative splicing in generating transcriptome diversity.^14^ A genome wide analysis revealed that ∼18% of human genes use multiple promoters.^17^ Promoter switches change the nature of the first exon, and hence the 5′ TL, and this event has been linked to a number of human pathologies.^18^^,^^19^ This switch is rarely complete but it can be amplified by the selective recruitment of one of the TL variants onto polysomes, as occurs with the MDM2 gene in tumor cells.^20^^,^^21^ Therefore, by generating 5′ TL heterogeneity, which can be both tissue and celltype specific, alternative promoters regulate the protein readout, the proteome and ultimately the cellular phenotype.^16^ Indeed, in a transcriptome/translatome analysis using a glioblastoma model, the authors concluded that selective polysomal recruitment of specific mRNA populations could itself initiate and drive tumor formation.^22^

We have discussed uORFs as translational regulatory elements. However, transcriptome analysis has identified thousands of yet non-annotated small open reading frames (smORFs) with the potential to encode biologically active peptides or SEPs (smORF-encoded proteins/peptides) smaller than 100 aa.^23^^,^^24^^,^^25^^,^^26^ Detecting the products of smORFs, which are numerous and small, is technically not straightforward.^27^ However, it has been facilitated by ribosome profiling.^6^ This technique couples ribosome footprinting to high-throughput RNA-seq and provides quantitative information about ribosome density across a transcript. It has been used to identify alternative START/STOP sites, initiation from non-AUG codons, translational pausing/frame-shifting as well as expression from uORFs and alternative ORFs.^28^ A bioinformatics analysis of the ribosome profiling database revealed that 40% of long non-coding RNAs (lncRNAs) carry smORFs and are expressed in human cells.^29^ Another source of SEPs are the uORFs, with ∼35% of mRNA coding genes having uORFs that are expressed.^29^ These SEPs can act either *in-cis* to modulate downstream initiation events, or have distinct biological function(s).^7^^,^^30^ Stalling of ribosomes over the AUG-CDS start site can also cause queuing of scanning ribosomes within the 5′ TL. This can permit the expression of smORFs initiating on near-cognate codons with the potential to increase the SEP repertoire.^31^^,^^32^ Another interesting group are the *cis*-acting peptides that are responsive to environmental signals and have been coined “peptoswitches”.^33^

Both leaky scanning and reinitiation permit access to internal AUGs. When in-frame with the principle ORF these give rise to N-terminally truncated proteins.^34^^,^^35^ When positioned internal and out-of-frame (sometimes referred to as ioORF: internal overlapping ORF), they represent a second source of smORFs. About 4% of human mRNAs appear to express proteins from AUG codons downstream of the AUG-CDS^29^ and the number of these polycistronic or dual-coding genes has been steadily rising.^36^^,^^37^ In fact, the expression of biologically active proteins from ioORFs has been known for some time. It was described in mammalian viral systems as far back as the 1980’s.^4^^,^^38^^,^^39^ Since then, genomic and proteomic technologies have identified thousands of previously non-annotated mammalian dual-coding genes.^24^^,^^40^ These ioORFs encode not only SEPs (<100 amino acids) but also longer proteins referred to as alternative or alt-ORF products (>100 amino acids). Many of these alt-ORFs exert a biologically function within the cell. For example, within the ataxin-1 (ATXN1) transcript, a smORF starting 30 nt downstream of the AUG-ATXN1, and in the −1 reading frame, expresses alt-ATXN1.^41^ This alt-ATXN1 (185 aa), co-localises and interacts with Ataxin-1 within nuclear inclusions. More recent examples include, the alt-FUS (170 aa) an inhibitor of autophagy and mitochondrial function,^42^ alt-B2R (157 aa) a modulator of the bradykinin signaling pathway^43^ and MINAS-60 (130 aa) that plays a regulatory role in 60S ribosome assembly.^44^ The alt-RPL36 (148 aa) is a regulator of the PI3K-AKT-mTOR pathway.^45^ Unlike the previous examples, all of which probably employ leaky scanning to access the alt-AUG start codon, it is initiated from a GUG codon upstream and out-of-frame with the RPL36-AUG.

Concerning the smaller SEPs, their implications for the human proteome are beginning to be appreciated.^46^ The prion protein gene *PRNP* also expresses a novel 64–81 aa polypeptide (depending on species) from a smORF, referred to as Alt-PrP.^47^ It localizes at mitochondria, is upregulated by ER stress and proteasomal inhibition and was detected in human brain homogenates, primary neurons, and peripheral blood mononuclear cells. Despite their small size, SEPs can have essential biological functions.^23^^,^^24^ In mice, the Mln ioORF expresses a 46 aa SEP implicated in muscle contraction.^48^ In humans, a 24 aa long SEP called humanin, synthesized from a lncRNA, is involved in apoptosis, interacting with BAX (Bcl-2-associated X protein),^49^ and the MRI-2 smORF (69 aa) has been implicated in DNA repair.^24^^,^^50^ Intriguingly, it has been proposed that in general, the expression of SEPs may be coupled to the stress response, an observation that would tie it in nicely with the process of translational reinitiation.^51^ With regards to clinical medicine, a number of human cancer specific antigens are also derived from smORFs.^52^^,^^53^ Their expression reflects the change in the translational landscape that occurs with cellular transformation and they represent novel targets for immune based therapies.^54^

**Figure 1:**
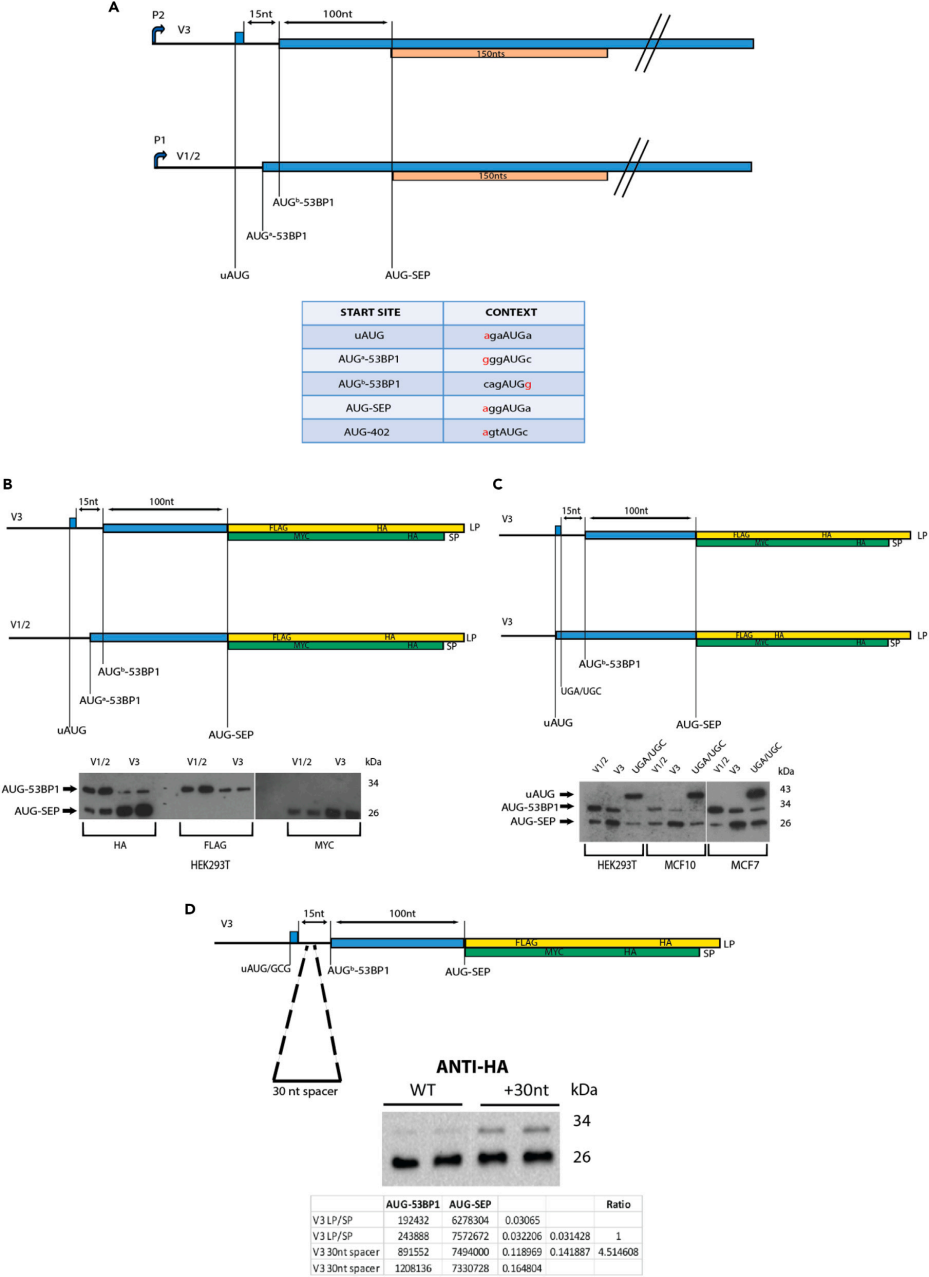
Reporter assays monitoring start site selection on the *TP53BP1* transcript variants (A) Schematic representation of the V1/2 and V3 transcript variants. P1 and P2 refer to the alternative promoters. The different AUG initiation sites are marked and there mammalian Kozak contexts^57^ are indicated in the lower table (nucleotides indicated in red indicate a positive context). The 53BP1 ORF is indicated in blue and the overlapping smORF in orange. The small blue rectangle in V3 refers to the uORF. (B) Upper panel: Schematic representation of the LP/SP reporter fused to the V3 and V1/2 sequences upstream of the AUG-SEP53BP1. The LP ORF (yellow rectangle) carries FLAG/HA tags and was fused in-frame to the 53BP1 ORF (indicated in blue). The AUG-SEP53BP1 started the SP ORF (green rectangle) which carries MYC/HA tags. These constructs were transiently expressed in HEK293T cells and protein steady-state levels were monitored on immunoblots using the anti-HA, anti-FLAG and anti-MYC Abs (lower panel). (C) Upper panel: Schematic representation of the V3 construct with the UGA/UGC mutation that fused the uORF to the 53BP1 ORF. The V1/2, V3 and V3UGA/UGC constructs were transiently expressed in HEK293T, MCF10A and MCF7 cells and steady-state protein levels were monitored on immunoblots using the anti-HA Ab that monitors expression from both overlapping ORFs (lower panel). (D) A schematic representation of the 30 nt spacer introduced between the uORF stop codon and the AUG-53BP1. This construct, and the WT, were transiently expressed in duplicate in HEK293T cells and expression monitored by immunoblotting using the anti-HA Ab. Band intensities were recorded and are indicated in the panel below the image. The intensity of the AUG^b^-53BP1 band was normalized to that of AUG-SEP53BP1 in each sample. The duplicate values were then averaged and used to evaluate changes in the relative ratio of the AUG-SEP53BP1 versus AUG^b^-53BP1 bands between both constructs.

In this manuscript, we have extended on our earlier study in which we reported a differential RNA-seq analysis on the tumoural MCF7 and non-tumoural MCF10 cell lines at both the level of the transcriptome and translatome.^55^ A number of genes were identified that exploited alternative promoters to generate 5′ TL heterogeneity that could, in-turn, modulate the protein readout. One of these, the *TP53BP1* gene, uses two promoters (Figure 1A). The P1 promoter (TSS12390) was active in both cell backgrounds. It generates two transcripts, referred to as V1 and V2 (NM_001141979.1,NM_001141980.1), which possess the same 5′ TL but differ because of an alternative splicing event within the CDS (hereafter referred to as V1/2). The second P2 promoter (TSS20205) was more active in MCF7 cells.^55^ It generates a V3 transcript (NM_005657.2) with a ∼278 nt 5′ TL carrying a 5-codon uORF whose stop codon is 15 nucleotides upstream of the AUG-53BP1 (Figure 1A). We postulated, and now confirm, that this uORF directs delayed reinitiation events at an internal smORF that expresses a 50 aa SEP which we refer to as SEP53BP1. The SEP53BP1 ORF is conserved in many (but not all) mammals. The endogenous SEP53BP1 protein has been detected in a number of human cell lines and shows punctate staining in both cytoplasmic and nuclear compartments. Sedimentation analysis suggests that it may have multiple intracellular partners and transient expression assays suggest that it “self-oligomerises”. Interactome studies using a yeast two hybrid (Y2H) approach indicate that the human protein interacts with components of the cellular protein turnover pathway, including the exposed C-terminus of the α4 subunit of the 20S proteasome barrel. Co-immunoprecipitation coupled to mass spectrometry (CoIP-MS) confirmed the interaction with the proteasome but also revealed an interaction with the TRiC/CCT (TCP1-ring complex or chaperonin containing TCP1) complex, an essential group II chaperone involved in the folding of up to 10% of the mammalian proteome.^56^ Thus, we have identified a novel small protein whose expression is linked to a promoter switch, coupled to a translational reinitiation event on an internal overlapping ORF. Its interaction with both the protein turnover machinery and cellular chaperones suggests that it may be playing a role in proteostasis. The study identifies *TP53BP1* as a new member of the dual-coding gene family.

## Results

### Organization and expression profiles of the TP53BP1 gene transcripts

In our earlier work, we reported on a differential RNA-seq analysis comparing the tumoural MCF7 and non-tumoural MCF10 cell lines.^55^ The *TP53BP1* gene was a particularly intriguing hit. It uses two promoters. The P1 promoter is active in both cell backgrounds. It generates two transcripts, named V1/2, originating from alternative splicing but carrying the same 5′ TL. The mRNA has two potential AUG start codons in the 53BP1 ORF, located at the end of the first and beginning of the second exons, and separated by four codons, hinting at two N-terminal isoforms (Figure 1A). These we refer to as AUG^a^-53BP1, which has a relatively good Kozak context, and AUG^b^-53BP1 whose context is poor (Figure 1A, lower panel). The 5′ TL is ∼113 nt long, 71% G/C and contains no uAUGs. The second P2 promoter was more active in MCF7 cells (Figure S1).^55^ Based on CAGE analysis it generates a V3 transcript with a ∼278 nt 5′ TL carrying a 5 codon uORF whose stop codon is 15 nucleotides upstream of the AUG^b^-53BP1 (Figure 1A: AUG^a^-53BP1 is in the first exon of the V1/2 transcript). Luciferase based reporter assays revealed that the V3 5′ TL was more repressive than V1/2 with regards to downstream initiation events because of the uORF.^55^ Furthermore, polysome gradient profiling of the two cell lines revealed that whereas the V1/2 transcript was mainly polysomal in both, the V3 transcript was polysomal only in the tumoural MCF7 cells.^55^ Therefore, at the outset of our current study we evaluated to what extent P2 promoter activity was a marker of the tumoural phenotype. We performed an RT-PCR and transcriptome analysis of V1/2 and V3 across a range of established tumoural and non-tumoural cell lines. No clear correlation with the tumoural phenotype was observed (Figure S1).

### The protein readout from the V3 mRNA is different from that of V1/2

The small uORF in the V3 5′ TL could promote delayed reinitiation events downstream of the AUG^b^-53BP1. Examination of the human sequence reveals that the next start codon downstream AUG^b^-53BP1 opens a smORF, +1 relative to the 53BP1 ORF, that would encode a polypeptide of 50 aa that we named SEP53BP1 (Figure 1A). To monitor expression in the V1/2 and V3 5′ TL backgrounds at both the AUG-53BP1 and AUG-SEP53BP1, we inserted the sequences upstream of the AUG-SEP53BP1 into our LP/SP overlapping ORF reporter.^10^^,^^58^ This fuses the 53BP1 ORF to LP (which carries an FLAG and HA tag) and the AUG-SEP53BP1 to SP (which carries an MYC and HA tag; the AUG-SEP53BP1 and its Kozak context were retained) (Figure 1B). The constructs allow us to follow initiation events at the AUG-53BP1 (we were unable to distinguish between the sites AUG^a^-53BP1 and AUG^b^-53BP1 on V1/2: however, based on context we presume that the former is the major start codon) (Figure 1A). Transient expression assays in HEK293 T cells, revealed that the V1/2 5′ TL directed initiation events mainly at AUG-53BP1 whereas with V3 the -majority of initiation events occurred at AUG-SEP53BP1 (Figure 1B). This pattern was also observed in transient assays performed in MCF10 and MCF7 cells (Figure 1C). To monitor the impact of the V3 uORF on the readout we mutated its stop codon (UGA→UGC: V3^UGA/UGC^) thereby fusing the uAUG to the 53BP1 ORF (Figure 1C). This effectively removes events arising from delayed reinitiation. When transiently expressed in HEK293T, MCF10 and MCF7 cells, the V3^UGA/UGC^ directed expression mainly from the uAUG (Figure 1C). This would be consistent with its good Kozak context (Figure 1A).

The results demonstrate high levels of initiation at the AUG-SEP53BP1 in transcripts carrying the V3 5′ TL and this is mediated by its small uORF. However, we wanted to ascertain to what extent reinitiation played a role in 53BP1 expression in the V3 context. For this, we introduced a 30 nt spacer element between the uORF and the AUG^b^-53BP1. Consistent with a reinitiation model, this increased initiation events at the AUG^b^-53BP1 relative to AUG-SEP53BP1 by between 4 and 5-fold (Figure 1D). This observation, in combination with the expression phenotype of the UGA/UGC mutant (Figure 1C), leads us to conclude that the majority of initiation events downstream of the V3 uORF arise by delayed reinitiation. Furthermore, the positioning of the short uORF close to a very leaky downstream AUG^b^-53BP1 means that initiation events occur mainly on AUG-SEP53BP1. This would direct the expression of the smORF-encoded peptide of 50 aa (SEP53BP1).^23^^,^^24^

### The configuration of the V3 5′ TL and the conservation of the smORF

**Figure 2:**
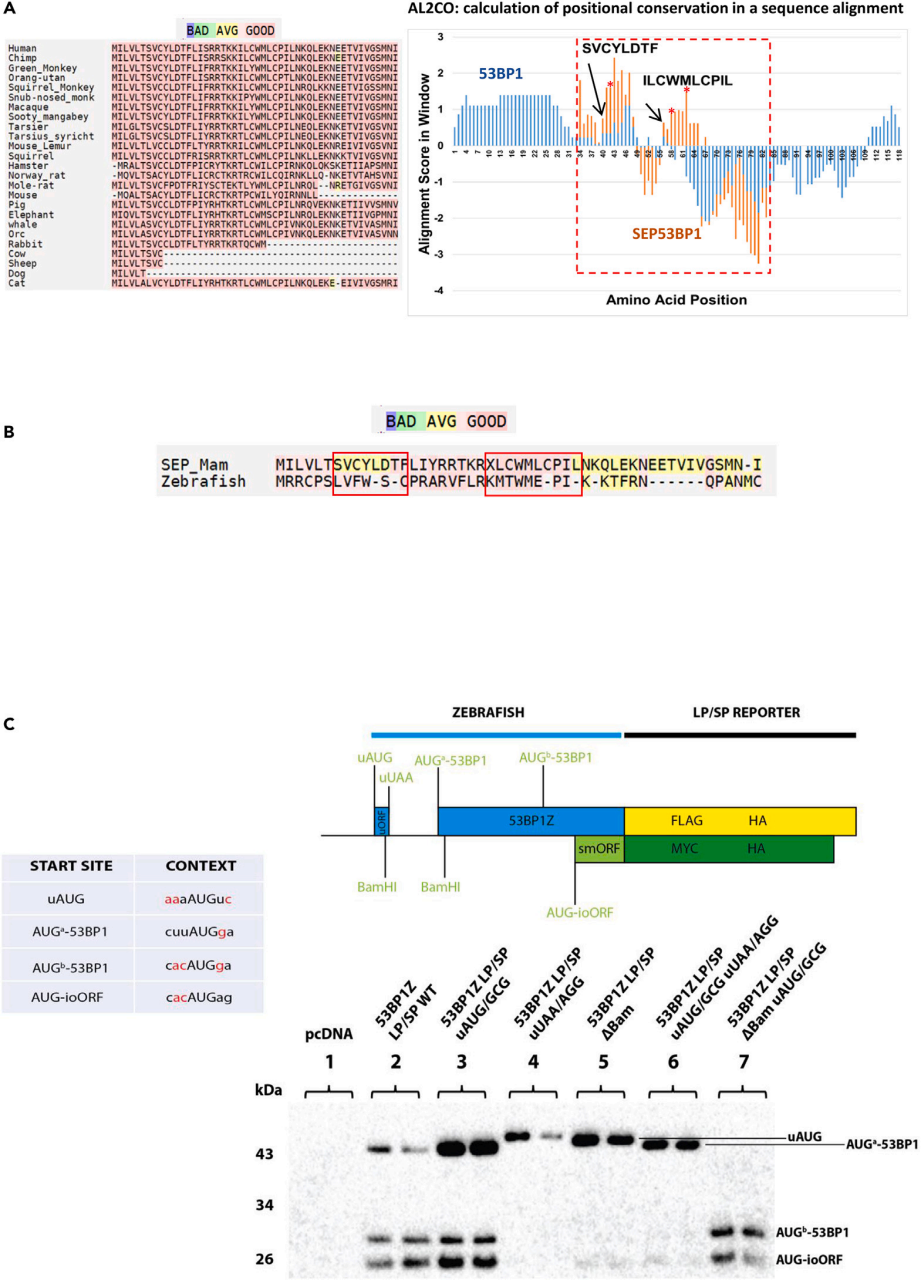
Delayed reinitiation mediated by a uORF promotes expression from an internal ioORF in *Danio rerio* (zebrafish) (A) Left hand panel: Alignment of smORF proteins encoded from an overlapping reading frame downstream of the AUG^b^-53BP1 in a range of mammals. The alignment was performed and scored using the T-COFFEE software (https://tcoffee.crg.eu) and is color coded from bad (blue) to average (yellow) to good (rose). Right hand panel: Starting from the ClustalW2 alignment across the N-terminal 118 codons of 53BP1 that encompasses the overlapping SEP53BP1 ORF (Figure S2), we performed a calculation of positional conservation using a sliding window of 8 aa with the AL2CO program.^61^ The alignment scores (arbitrary units) are plotted in blue for the 53BP1 ORF and light brown for the SEP53BP1 ORF (the region of which is delineated by the hatched rectangle). The primary sequence of the two highly conserved regions of SEP53BP1 is indicated, as are the positions of the three cysteine’s (red star). (B) Using the T-COFFEE software we generated a consensus mammalian SEP53BP1 sequence (Sep_Mam) and aligned it with the ioORF (smORF^Z^) observed in the zebrafish *TP53BP1* gene. Alignment scores are color coded (see above). (C) Upper panel: Schematic representation of the LP/SP reporter construct generated to monitor initiation events on the zebrafish transcript. Sequences downstream of the iORF stop codon (which was removed) were fused to the reporter, with the 53BP1 ORF (blue rectangle) fused to LP (yellow rectangle) and the smORF (light green rectangle) fused to SP (dark green rectangle). The position of the HA, FLAG and MYC tags are all indicated. The left hand table indicates the context for all the AUG initiation codons following the consensus rules established for zebrafish.^62^ Favourable nucleotides are highlighted in red. Lower panel: A series of LP/SP zebrafish constructs (as indicated above each lane) were transiently expressed in HEK293T cells. Immunoblots were performed with the anti-HA Ab. Each transfection was performed in duplicate.

The smORF responsible for SEP expression is conserved in most mammalian *TP53BP1* genes (Figure 2A), with the caveat that part of this conservation may arise from the constraints imposed by the overlapping 53BP1 ORF. Most of the key functional domains of the 53BP1 protein reside in its C-terminus and there may be more primary sequence plasticity within its long largely disordered N-terminus that can accommodate an overlapping ORF.^59^^,^^60^ Nonetheless, the SEP53BP1 ORF is found truncated in some mammalian species (in cow and sheep it is 9 codons and in dog it is 6 codons) despite the fact that the AUG-SEP53BP1 initiation codon tends to be conserved (Figure 2A: left hand panel). Starting from a ClustalW2 alignment across the N-terminal 118 codons of 53BP1, a region that encompasses the overlapping SEP53BP1 ORF, and selecting mammalian sequences that have retained an extensive smORF (Figure S2), we performed a calculation of positional conservation using a sliding window of 8 aa using the AL2CO program (Figure 2A: right hand panel).^61^ This revealed that whereas the positional conservation within the 53BP1 ORF dropped in the zone of the overlapping ORFs, the SEP53BP1 conservation was highly conserved in two blocks corresponding to the N-terminal 32 aa of the human protein (Figure 2B). This region would also cover the truncated mouse SEP53BP1 protein (Figure 2A). Most noticeable was the high conservation scores around the conserved cysteine’s (indicated as red stars in the figure) and tryptophan.

In the search for potential animal models of SEP53BP1 function and its regulation by a uORF (there is no uORF annotated for the mouse gene), we noted an extensive overlapping reading frame in the 5′ end of the *TP53BP1* gene in zebrafish (40 codons). Using the T-COFFEE software,^63^ we derived a consensus mammalian SEP53BP1 sequence and aligned this with the smORF sequence from zebrafish (Figure 2B). This gave good to average T-COFFEE alignment scores most noticeably within the N-terminal two-thirds (Figure 2B). Zebrafish have a single promoter expressing a 5′ TL variant with a uORF of 19 codons (GenBank: BC129236.1 and NM_001080170: longer than the human) whose stop codon is 15 nt upstream of the first AUG^a^-53BP1 (similar to human) (Figure 2C). As in the human V3 transcript, its AUG context is highly favourable according to the consensus rules established for zebrafish (Figure 2C).^62^ uORFs are frequently present in zebrafish transcripts and, as in mammals, they serve to modulate the translational readout.^11^ The zebrafish smORF (smORF^Z^) would express an “SEP-like” polypeptide (Figures 2B and 2C). The nucleotide spacing between the AUG^a^-53BP1 and AUG-ioORF is 400 nt in zebrafish compared to 97 nt in the human V3 mRNA. Within this 400 nt region there is a second AUG in the 53BP1 ORF (AUG^b^-53BP1) that could express an N terminally truncated (Δ121 aa) 53BP1 protein (Figure 2C). To examine initiation events on this transcript, we RT-PCR cloned all 53BP1 sequences upstream of the smORF STOP codon (changing it at the same time to a sense codon) starting from total zebrafish embryonic RNA. This was then fused to our LP/SP reporter to generate 53BP1ZLP/SP WT (Figure 2C). To monitor the role of the uORF on start site selection, a number of mutations were created. The uORF-AUG/GCG removed the start codon and the uORF-UAA/AGG fused the uORF to the 53BP1/LP ORFs in the reporter (Figure 2C). We also exploited two BamHI sites, one positioned just before the uORF UAA stop codon and the second just after the AUG^a^-53BP1. Deletion of the small BamHI fragment removed both the uORF^UAA^ and AUG^a^-53BP1 codons fusing uORF to the ORF of 53BP1/LP (Figure 2C: 53BP1ZLP/SPΔBam). As in the human reporter construct, the smORF was fused to the SP reading frame (Figure 2C). In the WT background, we could detect products from AUG^a^-53BP1, AUG^b^-53BP1 and AUG-ioORF, with the latter corresponding to the smORF^Z^ (Figure 2C, lane 2). Removal of the uAUG significantly enhanced expression at the AUG^a^-53BP1 but did not impact significantly on the downstream start sites (Figure 2C, lane 3). These latter initiation events would now arise because of leaky scanning through AUG^a^-53BP1 whose context is poor (Figure 2C). Thus as in humans, the uORF in zebrafish represses 53BP1 expression. Fusing the uORF to the 53BP1 ORF, either by the uORF^UAA/AGG^ mutation (Figure 2C, lane 4) or the ΔBamH1 deletion (which also removes AUG^a^-53BP1: Figure 2C, lane 5) produced a single band on the blot whose slower migration indicates that it arises from an initiation event on uAUG. The “non-leakiness” of this start codon would be consistent with its good Kozak context (Figure 2C). We confirmed this by introducing the uORF-AUG/GCG mutation into the ΔBamH1 background. The slow migrating band was lost and we restored the expression of products from the AUG^b^-53BP1 and AUG-ioORF (Figure 2C, lane 7). Thus, in zebrafish the uORF is also permitting initiation events downstream of the AUG-CDS (in this case AUG^a^-53BP1). These downstream initiation events can give rise to N-terminal truncated forms of the 53BP1 protein and the expression of smORF^Z^. However, unlike the human V3 transcript the configuration of the single zebrafish 5′ TL assures robust expression from all initiation sites (Figure 2C, lane 2). This may arise because all initiation sites downstream of the uORF have sub-optimal context sequences (Figure 2C).

Internal ORFs analogous to smORF^Z^ are observed in other fish species, for example carp (41 codons) and electric eel (58 codons). In this context, it is worth noting that despite the problems associated with 5′ TL annotation^64^^,^^65^ transcript variants with uORFs are annotated for both species. Carp (Ensembl database transcript ENSCCRT00000105636) has a uORF of 2 codons 47 nt upstream of the AUG-53BP1. Electric eel appears to have multiple promoters some of which generate 5′ TLs with uORFs, e.g., NCBI database transcripts XM_035520679.1 and XM_035520684.1.

### Transient expression of the human SEP53BP1 protein

**Figure 3:**
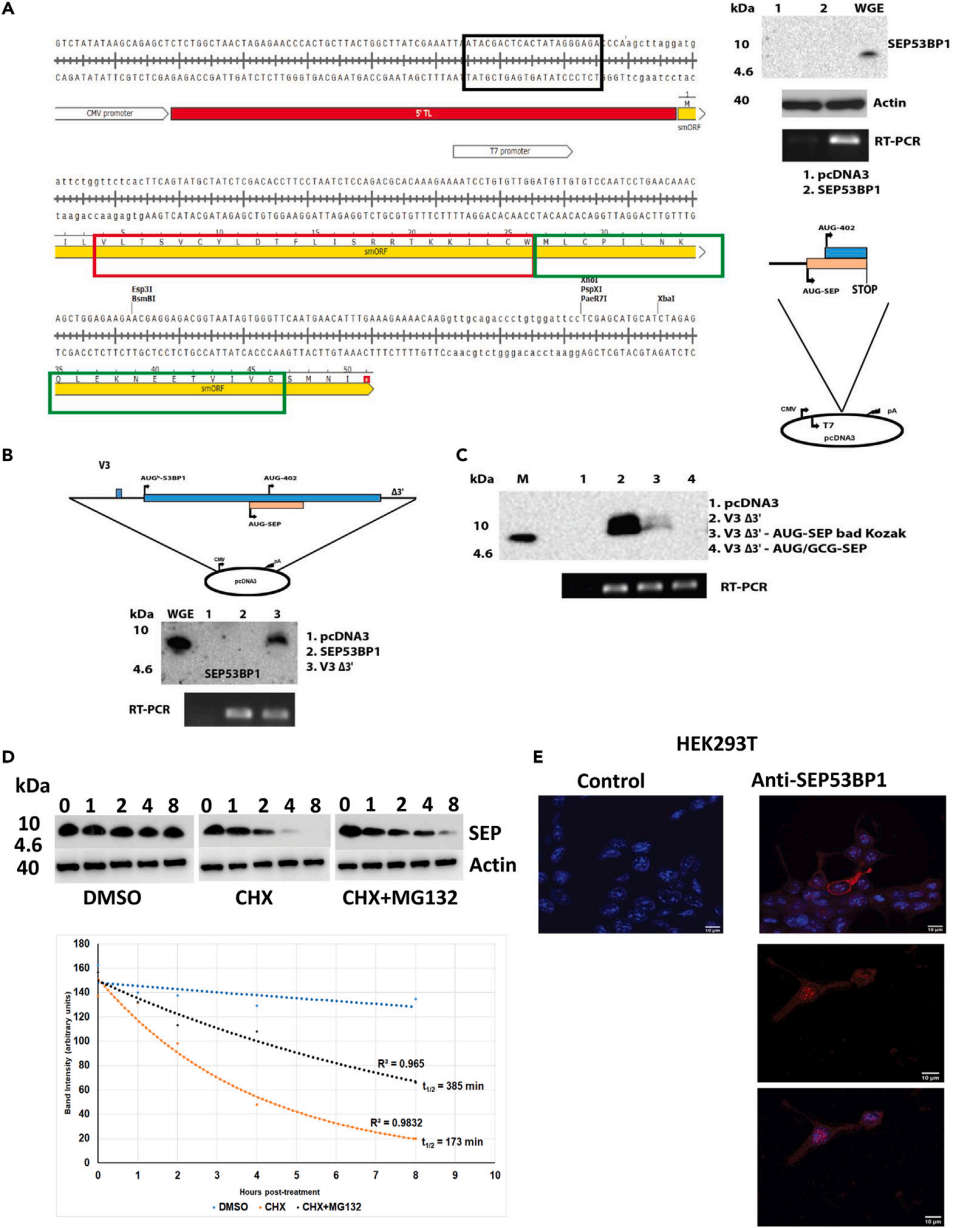
Transient expression assays with the human smORF expressing SEP53BP1 (A) Left hand panel: The coding region of the human smORF, with the AUG Kozak context retained, was inserted into a pcDNA3 expression vector under the control of the CMV promoter as indicated. The solid red region indicates the ∼70 nt 5′ TL derived from vector sequences and present on all transiently expressed mRNAs. The solid yellow rectangle is the smORF region with its amino acid sequence. Outlined with the red and green rectangles are the two peptides used to generate the polyclonal antibody. The position of the T7 promoter used to express *in-vitro* 5′ capped mRNAs is also indicated. Note that the 5′ TL is now much shorter. This construct, pcDNA3 smORF (indicated schematically in lower right image), and the parent empty vector, were transfected into HEK293T cells. Expression was monitored by immunoblotting using the anti-SEP53BP1 polyclonal Ab with an anti-actin loading control (upper right hand images). As a marker, a T7-generated capped smORF mRNA was translated *in-vitro* in a wheat germ extract (WGE). Transcript expression levels in the cell extracts was monitored by RT-PCR using a smORF-specific primer set. (B) All V3 sequences upstream of the smORF stop codon were cloned into pcDNA3 generating the clone V3Δ3’ (as shown schematically in the upper panel) and transiently expressed in HEK293T cells. Expression was monitored by immunoblotting using the anti-SEP53BP1 polyclonal Ab. M indicates an *in-vitro* generated SEP53BP1. Transcript expression levels in the cell extracts was monitored by RT-PCR. (C) Confirmation that the band observed in the V3Δ3′ transfected cells arose from initiation events at the AUG-SEP53BP1 was obtained by weakening the Kozak context (lane 3) and changing the AUG→GCG (lane 4). Transcript expression levels were monitored by RT-PCR (lower panel). (D) Using the V3Δ3′ construct, SEP53BP1 was transiently expressed in HEK293T cells seeded in a six well plate. At 16 h post-transfection cells were treated with DMSO (control) cycloheximide (CHX: 100 μg/mL) or CHX plus MG132 (10 μM) and recovered at the times indicated in the panel. The levels of SEP53BP1 and actin were monitored by immunoblotting. (E) IF studies using HEK293T cells transiently expressing SEP53BP1. The control was performed without the primary Ab. Nuclei were stained with DAPI (blue). The insert below the Anti-SEP53BP1 panel shows cells in which staining was mainly nuclear.

Polyclonal Abs against the SEP53BP1 protein were generated using two peptides that spanned most of the smORF (VLTSVCYLDTFLISRRTKKILC and WMLCPILNKQLEKNEETVIVG: Figure 3A). The Ab did not detect a SEP53BP1 protein in HEK293T cells (Figure 3A, lane 1), an observation that would be consistent with the low levels of the V3 transcript in this cell line (Figure S1). Using RT-PCR we amplified the smORF region, retaining the AUG-SEP53BP1 Kozak context (…agg**ATG**a…), and inserted this into a pcDNA3 vector. The CMV promoter driven expression would generate an mRNA transcript with a ∼70 nt 5′ TL derived from vector sequences (Figure 3A). However, transient expression assays using this smORF clone in HEK293 T cells failed to produce detectable amounts of protein despite robust transcription (Figure 3A). Furthermore, starting from the same cDNA clone we could express SEP53BP1 *in-vitro*, using a range of cell-free systems, programmed with T7 generated 5′ capped transcripts, despite their very short ∼18 nt 5′ TL (Figure 3A). To investigate this further, we generated a V3 clone containing all sequences upstream of the smORF stop codon that we named V3Δ3’ (Figure 3B). It should be noted that in all CMV-driven expression assays the mRNAs retain the same 5′ 70 nt vector sequence (see in upper panel of Figure 3A). Despite the presence of repressive translational elements upstream of the AUG-SEP53BP1 (namely, the uORF and the AUG^b^-53BP1: Figure 3B), a protein co-migrating with the *in-vitro* expressed SEP53BP1 was now observed in transient assays (Figure 3B). We confirmed that it arose from initiation events at the AUG-SEP53BP1 by both changing the AUG start codon to GCG and weakening its Kozak context (…agg**AUG**a … → … cgg**AUG**a…: this also confirmed the specificity of our Ab) (Figure 3C). The transiently expressed protein had a relatively short intracellular half-life (t_1/2_ = 173 min: Figure 3D). Curiously, inhibiting the proteasome with MG132 only partially stabilized it (t_1/2_ = 385 min: Figure 3D), suggesting the involvement of alternative turnover routes (e.g., autophagy) or protein loss via secretion.^66^ Immunofluorescence (IF) imaging of transfected HEK293 T cells revealed a mainly cytoplasmic localisation (Figure 3E). However, staining could be observed in the nucleus and, in rare occasions, it was almost exclusively nuclear (Figure 3E, lower panels).

### Nature of the sequences around the AUG-SEP53BP1 modulate start codon efficiency

**Figure 4:**
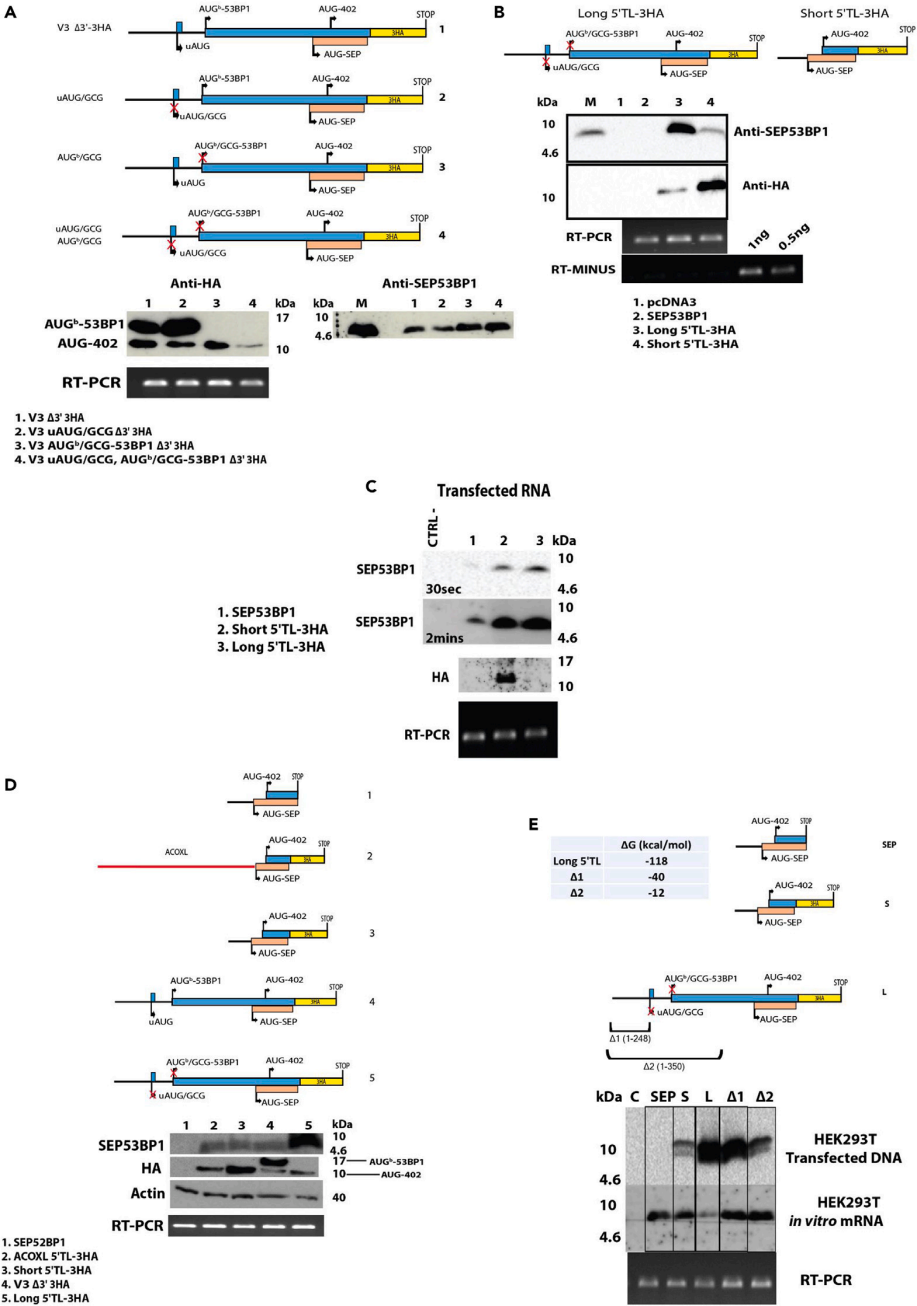
Sequences upstream of the smORF influence SEP53BP1 expression in transient assays (A) Starting from the V3Δ3′ backbone, we inserted a C-terminal 3HA tag into the 53BP1 ORF to monitor initiation events at AUG^b^-53BP1 and AUG-402 (schematically illustrated as construct #1). The different AUG codons upstream of AUG-SEP53BP1 were mutated to GCG as depicted schematically (clones #2–4). These clones were transiently expressed in HEK293T cells and expression monitored by immunoblotting using the anti-HA and anti- SEP53BP1 Abs (lower panels). Transcript levels were monitored by RT-PCR (lower image). (B) The Long and Short 5′ TL-3HA constructs are depicted schematically in the upper panels. These clones were transiently expressed in HEK293T cells and expression monitored by immunoblotting with the Abs indicated. The CTRL is an extract from V3Δ3′ transfected cells. Transcript expression levels in the cell extracts was monitored by RT-PCR. The RT-minus control is included as are RT-PCR reactions performed with known amounts of *in-vitro* generated transcript. This demonstrates that are “semi-quantitative” PCRs are in the linear range. (C) Using the T7 promoter we generated 5′ capped/polyadenylated transcripts for the smORF, Long and Short 5′ TL-3HA. These were transfected into HEK293T cells and expression monitored by immunoblotting. Two time exposures of the SEP53BP1 blot are shown. Intracellular mRNA levels were monitored by RT-PCR. (D) The different constructs schematically represented in the upper panels were transfected into HEK293T cells and expression monitored by immunoblotting using the anti-SEP53BP1, anti-HA and anti-actin Abs. Transcript levels were measured by RT-PCR using a primer set within the smORF. (E) The smORF (sm), Short (S) and Long (L) 5′ TL-3HA constructs are schematically represented in the upper panels. Below the L construct are indicated the two 5′ deletions (Δ1 and Δ2) that were generated, with the deleted nt sequences indicated in brackets. Inserted are the estimated free energies for the 5′ TL sequences calculated using the RNAfold web server (http://rna.tbi.univie.ac.at/cgi-bin/RNAWebSuite/RNAfold.cgi). These constructs were either transiently expressed in HEK293T cells or T7-generated capped mRNAs were used to program HEK293T cell extracts. SEP53BP1 expression was monitored by immunoblotting. The lower panel is an RT-PCR performed on extracts from the transiently transfected cells using a smORF primer set. The control (C) was either pcDNA3 transfected cell extracts or mRNA-minus *in-vitro* extracts.

We initially examined if SEP53BP1 expression from the V3Δ3′ was coupled to the presence of upstream initiation events. To monitor all initiation sites on V3Δ3′, including at a second AUG codon in the 53BP1 ORF located downstream of AUG-SEP53BP1 (AUG-402), we fused a 3HA tag to the latter’s C-terminal (V3Δ3′-3HA: Figure 4A construct #1). We then mutated the AUG codons upstream of AUG-SEP53BP1 to GCG both uniquely and in groups. The protein readout was monitored using anti-HA and anti- SEP53BP1 Abs. Mutation of the uAUG (uAUG/GCG) increased initiation at the downstream AUG^b^-53BP1, consistent with the repressional nature of the uORF, and marginally reduced initiation events further downstream, at AUG-SEP53BP1 and AUG-402 (Figure 4A construct 2). These latter events probably reflect the poor context of the AUG^b^-53BP1, which permits leaky scanning (Figure 1A). The GCG^53BP1(b)^ (construct 3) and GCG^uORF^/GCG^53BP1(b)^ double mutant (construct 4: hereafter referred to as Long 5′ TL-3HA) gave very similar expression profiles with the notable exception of the AUG-402 whose product levels were reduced in the latter. This would suggest that its utilization is also coupled to delayed reinitiation. Such a mechanism would allow the 40S to bypass the AUG-SEP53BP1 whose context is relatively good (Figure 1A). Furthermore, the double mutant indicates that the SEP53BP1 levels observed in the V3Δ3′ transient assays is not coupled to upstream initiation events, and a putative co-translational folding phenomenon, as we had initially hypothesised.

We noted that to detect SEP53BP1 in transient expression assays, one needed to include the 5′ upstream gene sequences even in the absence of any open reading frames (Figures 3B and 4A). We repeated this study, comparing the expression profiles of the Long 5′ TL-3HA and a short form that carried the same vector-derived 5′ TL sequences as smORF (Figure 3A). As observed previously, the presence of the Long 5′ TL significantly increased SEP53BP1 steady-state expression levels compared to the Short 5′ TL (Figure 4B). However, initiation at the downstream AUG-402 behaved in an inverse manner, indicating that in the short 5′ TL construct the AUG-SEP53BP1 was highly “leaky” despite the fact that the Kozak context was identical in both backgrounds (Figure 4B). This difference in AUG-SEP53BP1 efficiency was less noticeable when cells were transfected with 5′capped/polyadenylated mRNAs generated *in-vitro* from the same plasmid constructs (Figure 4C). However, the AUG-SEP53BP1 on the Short 5′ TL remained leaky, as evidenced by expression from the downstream AUG-402. Furthermore, the original smORF clone was barely expressed (Figure 4C). To explore this further, we asked if the effect of the Long 5′ TL resided in sequence specific elements or arose just because of length. We therefore substituted the Long 5′ TL with a sequence of similar length derived from the 3′ UTR of the *ACOXL* gene (407 nt). Its 68% G/C is the same as that found in the Long 5′ TL, the Kozak context remains unchanged and it contains no AUG codons. However, this construct produced SEP53BP1 levels equivalent to the Short 5′ TL in transient transfection assays, despite the fact that the AUG-SEP53BP1 appeared to be less leaky (Figure 4D: compare lanes 2 and 3). This figure also illustrates the fact that the low levels of SEP53BP1 observed in the Short 5′ TL context is equivalent to the levels observed with the Long 5′ TL carrying the repressive upstream elements, namely uAUG and AUG^b^-53BP1 (Figure 4D: compare lanes 3 and 4).

We next proceeded to trim the 5′ sequences from the Long, deleting region 1–248 (Δ1), which is predicted to remove the major RNA structural elements as confirmed by the change in the ΔG, and region 1–350 (Δ2) (Figure 4E). These were tested both by DNA transfection in HEK293T cells and by *in-vitro* translation in HEK293 T cell extracts programmed with capped/polyadenylated transcripts. *In-vitro*, the Long 5′ TL was highly repressive for SEP53BP1 expression compared to both Δ1 and Δ2, which each produced similar levels of expression. This probably arises because of the RNA structural elements cited earlier.^67^ However, in the transfected cell, the Long construct once again produced the highest levels of SEP53BP1 expression and this declined as one moved through Δ1, Δ2 and then the Short 5′ TL (Figure 4E). Therefore, the specific stimulatory effect of the Long 5′ TL, which seems to act on AUG-SEP53BP1 start codon efficiency and “non-leakiness”, is lost *in-vitro* and attenuated when the mRNA is transfected directly into the cell. This might suggest that the phenomenon is coupled to a nuclear-event as has been reported for cellular IRESes.^68^^,^^69^ However, in transiently transfected cells we failed to observe “IRES-like” activity within the Long 5′ TL using a simple bicistronic assay (Figure S3).

### Detection and localization of the endogenous SEP53BP1 protein

**Figure 5:**
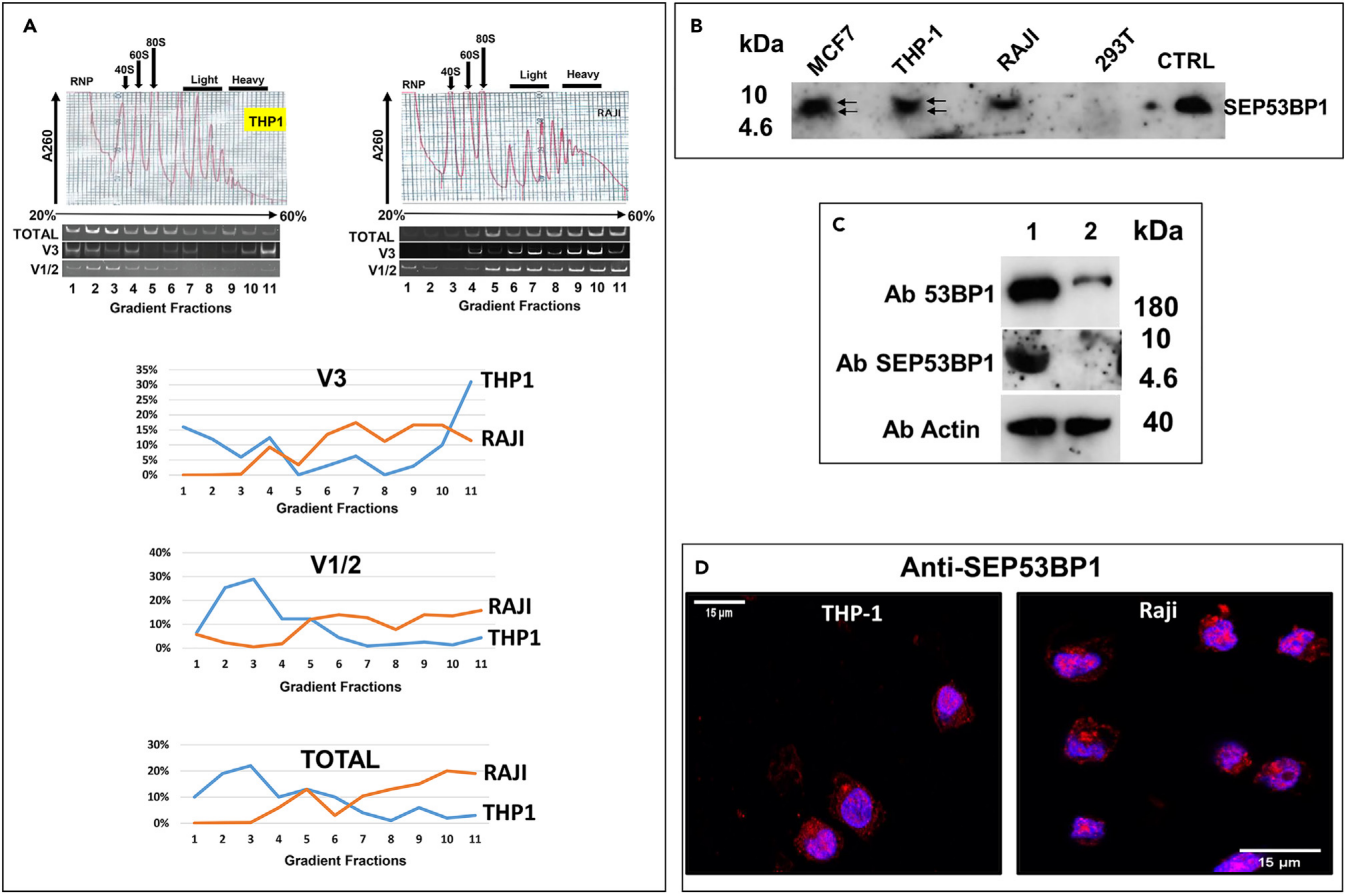
Detecting the endogenous SEP53BP1 protein (A) Upper panel: Polysome gradient profiles from THP-1 and Raji cells indicating the RNP (ribonucleoprotein), 40S, 60S, 80S, light and heavy polysomal fractions. Gradients were collected as eleven fractions. RT-PCR was performed on equal amounts of RNA from each fraction using primer sets specific for each variant and the total 53BP1. Amplicons were resolved on polyacrylamide gels (panels below the profiles), quantitated and plotted graphically as a percentage of the total signal for each variant (lower images). (B) Approximately 3x107 cells (MCF7, THP-1, Raji and HEK293T) were lysed in CSH buffer and immunoprecipitated with anti-SEP53BP1 Ab. Immune-complexes were recovered on Protein-G magnetic beads and analyzed by immunoblotting using the same Ab. The CTRL is the SEP53BP1 protein expressed *in-vitro*. The arrows in the MCF7/THP-1 lanes highlight doublet bands. (C) MCF7 cells (3x10^7^) were transfected with a scrambled siRNA (lane 1) or a siRNA targeting the *TP53BP1* gene (lane 2). Immunoblotting to observe both 53BP1 and actin was performed directly on the CSH cell extracts. However, the immunoblots to detect SEP53BP1 were performed after immunoprecipitation of the remaining cell extract as in (B). (D) Immunofluorescence images in THP-1 and Raji cells using the SEP53BP1 Ab (red) superimposed on the cell nuclei (blue). Images were generated by confocal microscopy.

Transient expression assays have allowed us to elucidate the mechanism by which *TP53BP1* P2 promoter activity will permit the expression of a novel SEP. However, at this point in the study it was necessary to detect the endogenous protein, and determine the cellular compartment(s) in which it accumulates as a route toward function. We had already observed that V3 transcript levels are regulated in a cell-specific manner (Figure S1). Furthermore, we had reported that polysomal recruitment of V3 could also be cell specific.^55^ With this in mind, we further scanned the ribosome-profiling database (http://sysbio.sysu.edu.cn/rpfdb/index.html). The image in Figure S4 was extracted from a study performed by the Brosch lab using THP-1 cells (a human acute monocytic leukemia cell line: https://www.ncbi.nlm.nih.gov/geo/query/acc.cgi?acc=GSE39561).^70^ The accumulation of P-site reads at the AUG-SEP53BP1 would be consistent with its utilisation as a start site.^71^ We therefore performed polysomal analysis of the total, V1/2 and V3 mRNAs in this cell background (Figure 5A). Only a minor fraction of the total 53BP1 gene transcripts were polysomal (26%) (Figure 5A, left hand profile). Concerning V3, very little was associated with light polysomes (9%) although a more significant fraction was observed within the heavy polysomes (44%), in particular the heaviest fraction (fraction 11, 32%). It is worth remembering that the smORF is only 150 nt in length and can accommodate a maximum of five elongating ribosomes.^6^^,^^72^^,^^73^ This would mean that V3 transcripts in the heavy polysomal fraction, that we define as >5 ribosomes per transcript, must be translating both ORFs. We also analyzed a human B lymphoblastoid cell line that was available to us, namely Raji cells (Figure 5A, right hand profile: no ribo-profiling data is available for this cell line). The polysomal profiles indicated that the majority (80%) of the 53BP1 transcripts were polysomal and this was also observed with both V1/2 (78%) and V3 (87%) (Figure 5A). Immunoblots detected SEP53BP1 expression in both these cell lines and in MCF7 cells, the cell line in which we originally reported V3 expression (Figure 5B).^55^ Curiously, in MCF7 and THP-1 cells, doublet bands were detected (indicated by the arrows in Figure 5B), reminiscent of the doublets that we sometimes observed with the transiently expressed protein (Figure 4E), and suggesting that post-translational modifications may be occurring. It has to be noted that detection of the endogenous protein required us to start with a large number of cells (>10^7^) with a subsequent enrichment by immunoprecipitation before immunoblotting. This probably reflects a low intracellular concentration combined with the technical difficulties associated with the detection of small proteins by immunoblotting.^74^ We confirmed that the band observed in MCF7 cells was expressed from the *TP53BP1* gene using a previously published siRNA whose target site overlaps the SEP smORF (Figure 5C).^75^ Confocal imaging of the endogenous protein in both THP-1 and Raji cells revealed punctate staining in both the nucleus and cytoplasm (Figure 5D). Z-stacking analysis confirmed the presence of endogenous SEP53BP1 in the nuclear compartment (see Videos S1 and S2 for THP-1 and Raji, respectively).

Video S1. Cellular distribution of endogenous SEP53BP1 in THP 1 cells, related to Figure 5D Z stacking was performed using a Zeiss LSM 800 confocal scanning microscope equipped with a Plan Apochromat 63 x/ 1 40 Oil DIC M 27 objective. For the THP 1 cells, 38 slices were taken (7.77 μm), in a manner that assured that the cell was imaged in its entirety. Pictures were analyzed using the Imaris software. The first few seconds of each video shows the original pictures collected, with the endogenous SEP53BP1 staining red and the nuclei staining blue. This was followed by a 3D reconstruction of the nucleus using the surface function within Imaris. In this representation, the cytoplasmic SEP53BP1 stains magenta and nuclear SEP53BP1 stains light blue.

Video S2. Cellular distribution of endogenous SEP53BP1 in Raji cells, related to Figure 5D Z stacking was performed using a Zeiss LSM 800 confocal scanning microscope equipped with a Plan Apochromat 63 x/ 1 40 Oil DIC M 27 objective. For the Raji cells, 30 slices were taken (6.09 μm), in a manner that assured that the cell was imaged in its entirety. Pictures were analyzed using the Imaris software. The first few seconds of each video shows the original pictures collected, with the endogenous SEP53BP1 staining red and the nuclei staining blue. This was followed by a 3D reconstruction of the nucleus using the surface function within Imaris. In this representation, the cytoplasmic SEP53BP1 stains magenta and nuclear SEP53BP1 stains light blue.

**Figure 6:**
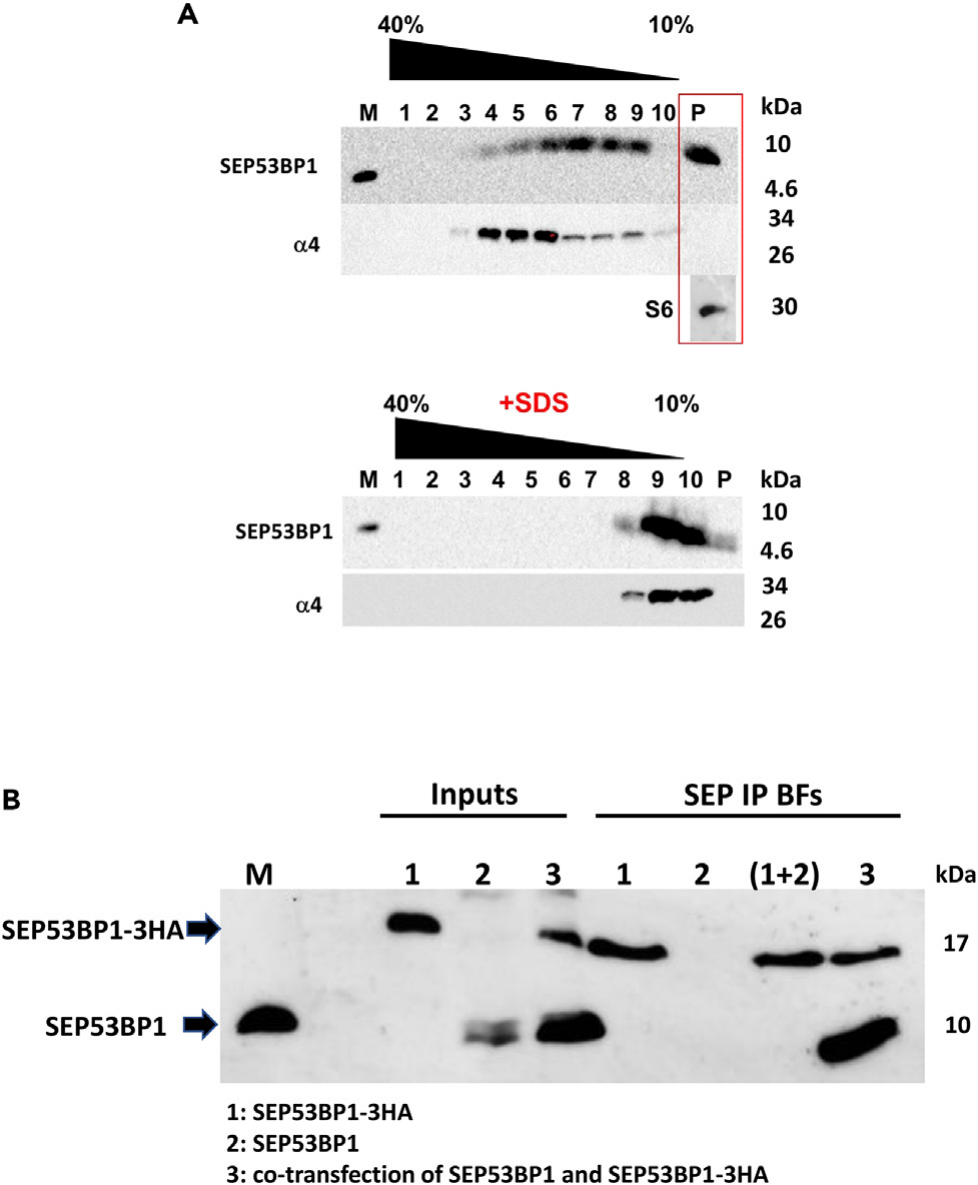
Intracellular behavior of the human SEP53BP1 protein (A) Glycerol gradient profiles generated from HEK293T cells transiently expressing SEP53BP1. The cell extracts were prepared in hypotonic lysis buffer supplemented with ATP (see STAR Methods). Ten equal fractions plus the pellet (P) were collected and probed for the SEP53BP1 and α4 proteins by immunoblotting. The pellet was also analyzed for the ribosomal S6 protein. In parallel, lysates were treated with SDS and heated at 65°C before loading onto the gradient (lower panels). M indicates a marker for the SEP53BP1 protein. (B) To evaluate if the SEP53BP1 protein can form intracellular multimers we transiently expressed SEP53BP1 and SEP53BP1-3HA either alone or together in HEK293T cells. Extracts were prepared in CSH buffer. Equal volumes of the singly transfected cells were also mixed. Singly transfected, dual transfected and mixed extracts were immunoprecipitated with the anti-HA Ab and the products analyzed by immunoblotting using the anti-SEP53BP1Ab. M indicates a marker for the SEP53BP1 protein.

The punctate staining in the cell suggested that the SEP53BP1 was interacting with intracellular structures and/or assembling into aggregates. To explore the former, we performed a sedimentation analysis using cytoplasmic extracts prepared from HEK293 T cells transiently expressing SEP53BP1. It revealed that despite its small size (50 aa); undetectable amounts remained in the upper fraction (fraction 10). A significant amount smeared through fractions 9 to 4, with fractions 4–6 corresponding to the 20S/26S zone based on the sedimentation of the α4 subunit of the proteasome (Figure 6A, upper panel). Extracts contained ATP to ensure 26S proteasome integrity during the assay.^76^^,^^77^ Even more striking was the presence of SEP53BP1 in the pellet fraction that included ribosomal subunits, as indicated by the presence of S6 (Figure 6A, upper panel). That this behavior corresponded to the formation of complexes was confirmed by SDS treatment of the extracts before gradient loading. (Figure 6A, lower panel). Overall, the sedimentation profile of SEP53BP1 is quite remarkable considering its small molecular size. Furthermore, it suggests that it may have multiple interacting partners in the cell.

To investigate multimerisation, we transiently expressed tagged (3HA) and untagged versions of SEP53BP1 either alone or together. We observed that on co-expression we could IP the untagged version of SEP53BP1 with the HA Ab, suggesting multimer formation (Figure 6B). This only occurred on co-expression of both forms and was not observed if individual tagged/untagged extracts were simple mixed before Ab addition (Figure 6B). Furthermore, in the co-transfected cells we expressed considerably more of the untagged versus the tagged protein (Figure 6B: lane 3 Inputs). This relative ratio was conserved after the HA Ab pull-down, suggesting that transiently expressed SEP53BP1 may be forming large multimers in the cell (Figure 6B: lane 3 Anti-HA pull-down). The formation of large and heterogeneous oligomeric complexes may also in part explain the sedimentation profile that we observed (Figure 6A).

### The SEP53BP1 interactome

**Figure 7:**
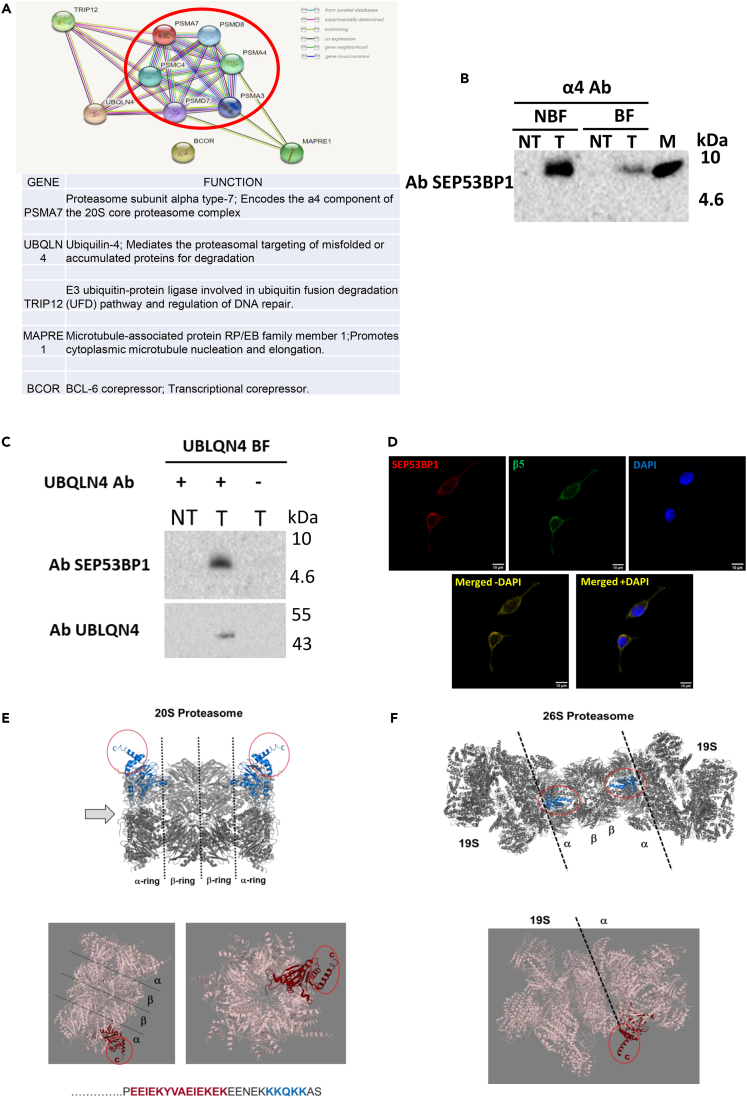
The SEP53BP1 interactome: Y2H analysis (A) String interactions (STRING software) generated using the five SEP53BP1 hits from the Y2H analysis as listed in the table. Circled in red are subunits of the proteasome. (B) Immunoblot analysis of the co-IP assay performed on extracts prepared from HEK293 T cells transiently expressing SEP53BP1 (T) or non-transfected controls (NT). Complexes were recovered on Protein-G magnetic beads carrying the α4 Ab and analyzed by immunoblotting with the SEP53BP1 Ab. NBF and BF indicate the non-binding and binding fractions from the pull-down, respectively. The CTRL is a marker for SEP53BP1. (C) Co-IP analysis performed using HEK293T cells transiently expressing both SEP53BP1 and UBLQN4 (indicated as T). Non-transfected cells (NT) served as a control. Cell extracts were prepared in CSH buffer and complexes were recovered on Protein-G magnetic beads alone (−) or beads carrying the UBQLN4 antibody (+). Beads were washed and resuspended in protein sample buffer before analysing by immunoblotting with SEP53BP1 and UBLQN4 specific Abs. (D) HEK293T cells transiently expressing the SEP53BP1 protein were grown and fixed on glass coverslips. The localisation of SEP53BP1 (red staining: upper left hand panel) and the β5 subunit of the proteasome (green staining: upper right hand panel) were analyzed by confocal IF microscopy using the Zeiss LMS800 confocal scanning microscope. A merged non-contrast-adjusted image is shown in the lower frame with the co-localisation signal indicated in yellow. The nuclei have been stained with DAPI (blue). (E) Upper panel: Side view of the 20S proteasome with the α4 subunits indicated in blue. The exposed C-termini are circled in red. The black dotted lines mark the interface between the α_7_β_7_β_7_α_7_ rings that form the 20S barrel. Lower panels: Rotations of the 20S barrel with α4s indicated in burgundy and the exposed C-termini circled in red. The orientation of the view on the right (which looks down the 20S barrel) is indicated by the arrow in the upper panel image. The amino acid sequence below is the C-terminal 27 aa of α4. In burgundy is the sequence that forms the extended α-helix visible in all α4 images on the proteasome, and in blue a putative nuclear localisation signal. (F) Upper panel: Side view of the 26S proteasome with the α4 subunits indicated in blue. The dotted black line marks the interface between the 20S cylinder and the 19S regulatory caps. Lower panel: Rotation of the 26S showing a 19S-α ring interface (black dotted line) with the exposed C-terminus of α4 circled in red. All images were extracted from the PDBe database (https://www.ebi.ac.uk/pdbe/node/1).

To gain insights into function we employed a yeast-2-hybrid (Y2H) screen to identify partners. SEP53BP1 was used as a prey, and screened against a peptide library generated from a human B cell Lymphoma_RP1. Around 51 million interactions were tested and 5 genes (PSMA7, UBQLN4, TRIP12, MAPRE1, BCOR) gave interactions with good confidence levels (Figure 7A). The selected interaction domain (SID) for each prey is depicted in Figure S5. String analysis connected four of the five genes (PSMA7, UBQLN4, MAPRE1, TRIP12) to proteasome biology (Figure 7A). We sought to biochemically validate this analysis focusing on the protein products of the first two genes. PSMA7 encodes the α4 subunit of the 20S proteasome barrel and it plays a key role in its assembly.^78^^,^^79^ We directly demonstrated the α4-SEP53BP1 association by co-IP performed on HEK293 T cell extracts transiently expressing the latter (Figure 7B). We noted that only a fraction of the transiently expressed SEP53BP1 co-IPed with the endogenous α4. However, this would be consistent with the sedimentation profiles of both proteins (Figure 6A). UBQLN4 also plays a role in the regulation of intracellular protein degradation by mediating the proteasomal targeting of misfolded or accumulated proteins.^80^ Its over-expression, as observed in some human tumors, also represses homologous DNA repair.^81^ We were unable to co-IP transiently expressed SEP53BP1 by pulling down the endogenous UBQLN4 protein. Nonetheless, we could pull-down SEP53BP1 with a UBLQN4 antibody when both were transiently over-expressed (Figure 7C). The necessity to over-express both to observe a co-IP signal may indicate that the interaction is weak and transient or that only minor sub-populations of each actually interact. Because α4 forms part of the 20S proteasome barrel we demonstrated intracellular co-localization of transiently expressed SEP53BP1 with the entire proteasome using antibodies against another subunit of the 20S, the β5 subunit of the β ring (Figure 7D). The co-localization signal was observed throughout the cell but mainly in what appears to be a cytoplasmic compartment.

**Figure 8:**
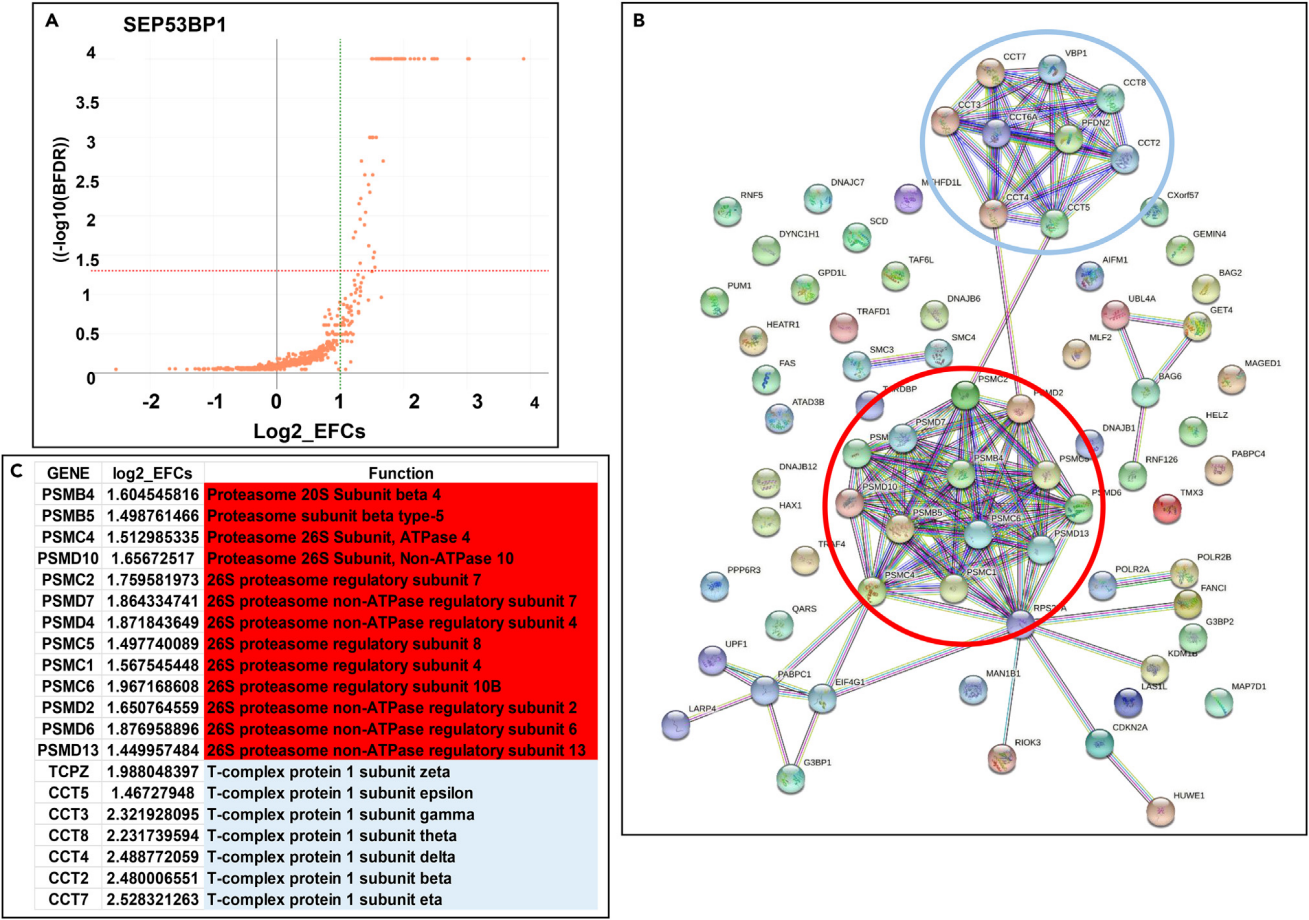
The SEP53BP1 interactome: CoIP-MS (A) Volcano plot showing −log_10_ transformed Bayesian false discovery rate (BFDR) as a function of log_2_ transformed effect size. The red dotted line shows the −log_10_-transformed BFDR threshold of 0.05, while the dotted green line gives the log_2_ transformed empirical fold-change (EFC) of 2. Promising candidate pray proteins are found in the upper right sector of the plot. (B) String interactions (STRING software) generated using the seventy-four SEP53BP1 hits from the coIP-MS analysis (Table S2). The two major nodes are indicated: red circled are subunits of the proteasome and green circled the TRiC/CCT chaperonin complex. (C) A list of the proteasome and TRiC/CCT complex protein subunits identified in the coIP-MS (colored coded to match the circles in B).

To further probe the interactome, we performed CoIP-MS starting from HEK293T extracts transiently expressing SEP53BP1-3HA. In total, 945 proteins (min. 2 peptides per protein) were identified and quantified using intensities across all samples. After subtraction of the empty vector control and further data mining, we obtained 74 hits (Figure 8A, Table S2). String analysis again revealed that the interactome ofSEP553BP1 included the proteasomal pathway (Figures 8B and 8C). However, another hit not observed in the Y2H analysis, was the TRiC/CCT (TCP1-ring complex or chaperonin containing TCP1) complex, an essential group II chaperone involved in the folding of up to 10% of the mammalian proteome (Figure 8B).^56^ This chaperone is composed of eight subunits that form a ring and our mass spec analysis identified seven of these (Figure 8C).

## Discussion

It is increasingly evident that the complexity of the metazoan proteome is considerably increased by the expression of SEPs (<100 aa), which until recently escaped detection using conventional biochemical procedures. They are also referred to as small protein,^82^ alt-ORF,^83^^,^^84^ nORF,^85^ miniproteins or microproteins.^82^ The presence of these products, encoded by smORFs, was initially predicted by Basrai and coworkers,^86^ and has subsequently been confirmed using techniques such as ribosome profiling, proteogenomics and conservation signatures.^82^^,^^87^ The OpenProt database now lists nearly 21,000 human SEPs although it remains unclear if all these proteins are expressed. (www.openprot.org).^88^ They arise mainly from lncRNAs and uORFs.^24^^,^^89^ However, they also arise from internal overlapping ORFs or even the 3′UTR, bringing to an end the dogma that eukaryotic mRNAs are monocistronic.^25^^,^^41^^,^^46^^,^^47^^,^^50^^,^^90^^,^^91^^,^^92^ Furthermore, studies have ascribed diverse functions to SEPs, ranging from the regulation of physiological functions within the cell^82^^,^^93^ to crucial developmental functions within metazoan species separated across large evolutionary distances.^30^^,^^94^^,^^95^^,^^96^^,^^97^^,^^98^^,^^99^

In this manuscript, we have identified a new member of the SEP family expressed from a smORF within the *TP53BP1* gene, the main CDS of which expresses a protein that plays a central role in non-homologous DNA repair.^100^ Therefore, *TP53BP1* becomes a new member of the mammalian dual coding or polycistronic genes.^36^^,^^37^ The mode of SEP53BP1 expression is also novel. In humans, it couples alternative promoter activity (P1 versus P2) to a translational reinitiation event on the internal AUG-SEP53BP1 mediated by a short uORF within the P2 derived mRNA 5′ TL. Both these events can potentially respond to intracellular stresses.^101^^,^^102^^,^^103^ Intriguingly, in transient DNA transfection assays the efficiency of the AUG-SEP53BP1 (and it’s “non-leakiness”) is positively modulated by *TP53BP1* specific region(s) upstream of the SEP53BP1 smORF. This extends beyond the conventional Kozak consensus.^57^ Whereas it has been known for some time that RNA regions downstream of an initiation codon can modulate its efficiency, this is somewhat novel with regards to an upstream region.^104^ Its importance is highlighted by the fact that DNA expression plasmids carrying only the smORF fail to express SEP53BP1 in transient assays despite robust mRNA levels in the cytoplasm (Figures 3B and 3C). Exactly how the upstream sequence functions remains unclear but its effect is lost in cell extracts, and is only partially retained when mRNAs are transfected directly into the cell (Figure 4). This might suggest a “nuclear-event” as proposed for some cellular IRESes in which proteins recruited during pre-mRNA processing and transport, function as IRES *trans*-acting factors in the cytoplasm.^105^ However, a simple DNA-based bicistronic assay failed to demonstrate IRES-activity, despite the fact that such an approach tends to produce false positives (Figure S3).^106^ Another possibility is that the upstream region may possess properties similar to the cap-independent translational enhancers (CITEs).^107^ These are RNA sequence/structural elements downstream of the 5′ cap that promote binding of components of the translational machinery that in-turn facilitate ribosome binding. In a similar vein, the region upstream of the SEP53BP1 smORF may recruit factors (possibly during nuclear-cytoplasmic transport) that subsequently interact with the scanning ribosome to increase the efficiency, and non-leakiness, of the AUG-SEP53BP1. Alternatively, dynamic methylation events in the upstream region could be modulating AUGSEP53BP1 “leakiness”, a phenomena already observed in the ATF4 mRNA in which m^6^A modifications within in the 5′ TL control ribosome scanning and subsequent start codon selection.^108^

The number of annotated mammalian SEP and alt-ORF proteins has been steadily increasing thanks mainly to more robust proteomic and translatomic (i.e. ribo-profiling) analysis pipelines. However, one limitation of these approaches is that they provide limited if no information on the nature of the translational mechanism regulating the proteins expression. Rarely do these studies examine the nature of the regulatory elements within the 5′ TL, elements that are crucial particularly in the context of a dual coding gene in which startsite selection can be controlled. Compounded with this, is the complexity that arises from 5′ start heterogeneity and promoter switches that alter the nature of the 5′ TL.^16^ Concerning the expression from overlapping ORFs (or alt-ORFs), the context of the AUG-CDS (particularly its 5′ upstream sequences) and the presence of uORFs are all features that will regulate PIC access to the downstream AUG-alt-ORF. Based on current models, and the observation that ∼50% of human 5′ TLs contain one or more uORF(s) with a medium size of only 17 codons, leads one to propose that start site selection coupled to delayed initiation is a significant mechanism in alt-ORF expression.^82^ Features such as uORF number, length, position as well as the activity of key initiation factors would regulate the magnitude of the initiation event at the internal start site(s).^5^^,^^109^^,^^110^ Therefore, despite the power of ribo-profiling at predicting dual coding genes,^111^ the story of SEP53BP1 highlights many of the complexities associated with the identification of alt-ORFs. The expression of the V3 transcript (carrying the uORF and the major source of SEP53BP1) is low (<5% of the total 53BP1 mRNA) in most of the cell lines analyzed (Figure S1B). This observation would explain the low P-site read score in the ribo-profiling study in THP-1 cells (i.e. most of the reads map to the major V1/2 transcript that expresses mainly the CDS: Figure S4). Delayed reinitiation also couples the translational readout to cellular stress. Curiously, it has been proposed that SEP expression may be an integral part of the cellular “stress response”.^112^ The link to developmental functions is also intriguing, because promoter switching and translational reprogramming are key events during differentiation in metazoans.^113^^,^^114^ Extensive studies on SEP protein expression, and function, have been performed using model systems such as *D. melanogaster*,^94^^,^^96^^,^^97^^,^^115^ and riboprofiling studies have confirmed the presence of smORFs in zebrafish.^116^ Consequently, zebrafish presents itself as a useful animal model to explore the role of the uORF in *TP53BP1* gene expression in metazoans.

The human SEP53BP1 protein is predicted to have a highly ordered structure (Figure S6). As predicted for overlapping reading frames, the internal SEP53BP1 ORF overlaps a region in the 53BP1 ORF that is predicted to encode a portion of the 53BP1 protein that is largely disordered in structure (Figure S6).^60^ Furthermore, sedimentation profiles suggests that it may have multiple partners in the cell (Figure 6A). These studies were performed using only the cytoplasmic fraction, but IF images indicate that both the transiently expressed and endogenous protein is also located in the nucleus (Figure 3E, Animations S1/2). Therefore, the protein may have distinct partners/function(s) in each compartment. This functionality issue is rendered even more intriguing (and potentially complexed) by the observation that the human SEP53BP1 can multerimerize and may exist in multiple oligomeric forms each with a unique function(s).

As an avenue to function, we performed interactome studies using both a Y2H approach and coIP-MS. The Y2H study revealed that SEP53BP1 associates with components of the cellular protein turnover apparatus, including the α4 subunit of the 20S proteasome barrel (Figure 7). The SID on α4 maps to the C-terminus (Figure S5), a region that is found largely exposed on the surface of the 20S and 26S proteasome (Figures 7E and 7F). Interaction with components of both the 20S and regulatory 19S subunits was also observed in the co-IP-MS analysis (Figures 8B and 8C). These two subunits assemble to form the 26S proteasome that specifically targets and degrades polyubiquitinylated substrates in an ATP-dependent manner.^117^ This polyubiquitinylated selectivity resides within the 19S complex positioned at each extremity of the 20S cylinder (Figure 7F).^118^ However, during certain stresses, binding of activating proteins can open the α ring on the 20S permitting the entry of protein substrates. Proteins that enter are degraded in an ubiquitin/ATP independent manner. This “active” 20S serves to remove misfolded or oxidized proteins that accumulate during the stress.^119^ It remains to be determined if SEP53BP1 is modulating the assembly and/or the activity of the 26S and/or “active” 20S, both of which retain the α4 SID. Proteasomes are found in both cytoplasmic and nuclear compartments.^77^^,^^120^ One of the putative nuclear localization signals is actually located on the C-terminal tail of α4 (Figure 7E).^121^ It seems conceivable that the nuclear SEP53BP1 may enter in association with the proteasome. Furthermore, proteasome levels in the nucleus responds to stresses, such as glucose starvation, hypoxia or low pH^122^ many of which may also be modulating SEP53BP1 intracellular levels.

The coIP-MS analysis also revealed interactions with a number of heat shock proteins and cellular chaperones (e.g. DNAJB12, DNAJC7, DNAJB1, PFD2, BAG2: Table S2) and gave hits on seven of the eight subunits that constitute the ring of the essential TRiC/CCT chaperonin complex (Figures 8B and 8C). The transiently expressed SEP53BP1 protein is relatively unstable and a fraction (∼50%) appears to be degraded by the proteasome (Figure 3D), an observation that may in part explain the interaction observed in the coIP-MS analysis. However, the α4 interaction observed in the Y2H study is occurring independent of proteasome assembly suggesting that the SEP53BP1-proteasome interaction occurs independent of proteasome function. The complexed SEP53BP1 interactome, which includes multiple targets in the protein folding and turnover pathways, leads us to suggest that it may play a role in regulating intracellular proteostasis.

### Limitations of the study

The studies on the translational regulation of SEP53BP1 expression have been performed by transient over-expression using reporter constructs. Despite the fact that these novel reporters permitted monitoring of initiation events at multiple, out-of-frame sites, it remains to be determined if all observations apply to the endogenous mRNA whose intracellular levels are much lower. In addition, it is unclear how 53BP1 sequences upstream of the AUG-SEP53BP1 can actually improve the efficiency and non-leakiness of the start codon. The regulation of the alternative *TP53BP1* promoter, which drives the expression of the V3 transcript and is at the heart of SEP53BP1 expression, also remains obscure. Our imaging of the endogenous protein reveals that it is present in both nuclear and cytoplasmic compartments. However, in validating the Y2H interactome we have analyzed only the cytoplasmic fraction. Likewise, the study employing coIP-MS probed only the cytoplasmic interactome. Therefore, it remains to be determined if the protein partners are the same in each compartment. Furthermore, approaches to explore function, employing knock-down or knock-out strategies, are rendered technically complexed because of the overlapping ORF configuration. Although we are confident of our current interactome dataset, the consequence of SEP53BP1 expression on both proteasome and TRiC/CCT chaperonin complex assembly and/or activity is yet to be resolved. The establishment of a function(s) will also confirm that SEP53BP1 is not merely a neutral translational by-product that arises from the regulation of intracellular 53BP1 levels via the dual promoter configuration.

## STAR★Methods

### Key resources table

**Table:** 

REAGENT or RESOURCE	SOURCE	IDENTIFIER
**Antibodies**		

anti-53BP1	Santa Cruz	#sc-22760
anti-phospho (Ser51) eIF2α	GenTex	#61039
anti-eIF2α	Invitrogen	#44728G
anti-HA	Covance	clone 16B12
anti-FLAG	Sigma	M2 antibody
anti-actin	Millipore	#MAB1501
anti-RPS6	Cell Signaling	#2317
anti-A1Up (UBQLN4)	Santa Cruz	#sc-136145
anti-α4 (PSMA7)	Santa Cruz	#sc-58417
anti-β5	Santa Cruz	#sc-393931
goat anti-mouse/rabbit HRP	Bio-Rad	#1706515/6
Anti-MYC tag	University of Geneva	Prof. Dominique Soldati
Alexa Fluor 594 goat anti-rabbit IgG (H+L)	Invitrogen	#A27016

**Bacterial and virus strains**		

*E. coli* DH5α	ThermoFisher	#18265017

**Chemicals, peptides, and recombinant proteins**		

Lipofectamine 3000	Invitrogen	#L3000001
DMEM	Gibco	#41966
DMEM F-12 medium	Gibco	#31331
penicillin/streptomycin	Gibco	#15140-122
fœtal bovine serum	Brunschwig	#F003221G
estradiol	Sigma	#E8875
horse serum	Brunschwig	#P300702 PAN
EGF	Sigma	#E9644
Dexamethasone	Sigma	#D8893
human recombinant insulin	Sigma	#I9278
2-mercaptoethanol	Gibco	#31350-010
1X amino acids	Gibco	#11140-035
sodium pyruvate	Sigma	#S8636
Sucrose	Sigma	#S0389
cycloheximide	Sigma	#C1988
NP-40	Applichem	#A1694
aprotinin	Merck	#A6106
PMSF	Merck	#329-98-6
RNasin	Thermofisher	#E00382
cocktail inhibitor	Roche	#04693132001
TriZol	Ambion	#15596018
Superscript II	Promega	#M3682
Pfu polymerase	Rovalab	#PF100
Acrylamide/bis (19:1)	Bio-Rad	#1610144
APS	Invitrogen	#C2005
TEMED	Sigma	#T9281
Tris base	Sigma	#T1503
Tricine	AppliChem	#A1085,0250
Tween-20	AppliChem	#A4974,0500
Triton X-100	AppliChem	#A1388,0500
β-mercaptoethanol	Bio-Rad	#1610404
SDS	Sigma	71729
bromophenol blue	Bio-Rad	#161-0404
Coomassie Blue G	SERVA	#42 655
Bradford Reagent	Cytoskeleton, USA	#ADV02
PVDF membrane	Millipore	#IPVH00010
Lysolecitin	Sigma	#L5254
ATP/GTP	NEB	#N2080A
creatine phosphate	Sigma	#27920
creatine kinase	Roche Sigma	#C3755
amino acid mix	Promega	#L447C
spermidine	Sigma	#85558
calf liver tRNA	Sigma	#R7250
DTT	Promega	#P117A
BSA	Promega	#R3960
DAPI stain	Invitrogen	#D1306
Prolong Diamond Antifade Mountant	Invitrogen	#P36961
RNAiMAX reagent	Invitrogen	#13778-075
Dynabeads Protein G	Invitrogen	#10004D
anti-HA magnetic beads	Pierce	#88836
mMessage mMachine™ T7 Transcription Kit	Invitrogen	#AM1344
vaccinia capping system	NEB	#M2080S
polyA tailing kit	Invitrogen	#AM1350

**Critical commercial assays**		

Wheat germ extracts	Promega	#L4380
Dual-Luciferase Reporter Assay System	Promega	#E1910
WesternBright™ Quantum	Advansta	#K-12042-D10

**Experimental models: Cell lines**		

HEK293T	ATCC	CRL-3216
HeLa	ATCC	CCL-2
MCF-7	ATCC	HTB-22
MCF-10	ATCC	CRL-10317
THP-1 and Raji	University of Geneva	Prof. Walter Reith

**Oligonucleotides**		

53BP1_iORF_LP_SP	This paper	AGTTCTAGAGGATGAGACTCAGCACGTCTCAACCACG
53BP1 V1V2+	This paper	GATCTGAATTCGAGTTCGCGGCCGGTGGCGG
53BP1 V3+	This paper	GATCTGAATTCGTTTTTTGTCACTGCCTGCC
V1_2+	This paper	TAAAGCTTGAGTTCGCGGCCGGTGGCGG
V1_2-ve	This paper	ATCTGCTCCCCAGCCATGGCGGCGGGAGGT
V3+	This paper	TAAAGCTTGTTTTTTGTCACTGCCTGCC
V3-ve	This paper	AGTAGGGTCCATGGGCTCCCCAGGGACTCA
53BP1 Tot+	This paper	CAATACTACACATTCCCTTGGTGC
53BP1 Tot-	This paper	GAGTTGGTGGTTACTGATTGTAGC
SPACER_30nt_uORF +ve	This paper	AGCACAGAGCCTCGCCTTTGCCGATC CGCCGTCCCTGGGGAGCAGATGGA
SPACER_30nt_uORF-ve	This paper	GGCGGATCGGCAAAGGCGAGGCTCT GTGCTTCAAGGCCGGAAGGTCATTC
V3 LP-SP AUG/GCG 53BP1	This paper	ACTTCCAGTAGGGTCCGCCTGCTCC CCAGGGAC
V3 53BP1-ve	This paper	GAAGTGAGAACCAGAATCATCCTCTA GAACCTGGCTTTCAGGC
SEP53BP1 XhoI-ve	This paper	GCCTCGAGGAATCCACAGGGTCTGCAAC
SEP53BP1 bad kozak-ve	This paper	ACTGAAGTGAGAACCAGAATCATCCG CTAGAACCTGGCTT
SEP53BP1 AUG/GCG +ve	This paper	TGAAAGCCAGGTTCTAGAGGGCGATTC TGGTTCTCACTTCAGTATG
SEP53BP1 AUG/GCG-ve	This paper	CATACTGAAGTGAGAACCAGAATCGCC CTCTAGAACCTGGCTTTCA
HindIII_UBQLN4 +ve	This paper	CTC AAG CTT GGC ATG GCG GAG CCG AGC GGG GCC GA
UBQLN4_XbaI-ve	This paper	GAG TCT AGA TTA GGA GAG CTG GGA GCC CAG CA
HindIII α4 +ve	This paper	CTCAAGCTTGGCATGAGCTACGA CCGCGCCATCA
Zebra ioORF_LPNext Xba(-)	This paper	GTTCTAGAGTACAGTGTGTGGT AGAGTTAGC
Zebra uAUG_GCG HIII(+)	This paper	GTTAAGCTTGTGCACTGTAAAGC GTCACACGTTCGC
Zebra_53BP1_5’HIII(+)	This paper	GTTAAGCTTGTGCACTGTAAAA TGTCACACG
Zebra_ioORF_RT	This paper	CTTCTGACCCGGTTGAATTTCCATC
Zebra_iORF_Xho(-)	This paper	CAACTCGAGGAACTTTTCAACAC ATATTGGC
siRNAs *TP53BP1*	This paper	UAUUACCGUCUCCUCGUUCTT and GAACGAGGAGACGGUAAUATT
scrambled siRNAs *TP53BP1*	This paper	GGUGCGCUCCUGGACGUAGCCTT and GGCUACGUCCAGGAGCGCACCTT

**Recombinant DNA**		

pcDNA3.1	Invitrogen	V79020

**Software and algorithms**		

ImageJ software	NIH	https://imagej.nih.gov/ij/
Image Lab software	BioRad	https://www.bio-rad.com
Imaris software	Oxford Instruments	https://imaris.oxinst.com
SaintExpress software	Significance Analysis of INTeractome	http://sourceforge.net/projects/saint-apms/files/
PONDR	Predictor of Natural Disordered Region	http://www.pondr.com
I-TASSER	Iterative Threading ASSEmbly Refinement	https://zhanggroup.org
AL2CO	Calculation of positional conservation in a sequence alignment	http://prodata.swmed.edu/al2co/al2co.php
STRING:	Functional protein association networks	https://string-db.org/
T-COFFEE software	Multiple sequence alignment server	https://tcoffee.crg.eu

### Resource availability

#### Lead contact

Further information and requests for resources and reagents should be directed to Joseph A. Curran (Joseph.Curran@unige.ch).

#### Materials availability

Plasmids generated in the study will be available upon request.

### Experimental model and subject details

#### Cell culture

HEK293T, HeLa, MCF7, MCF10A, THP-1 and Raji cells were grown at 37°C in a humidified 5% CO_2_ chamber. Cells were cultured in Dulbecco’s modified Eagle's medium (DMEM) supplemented with 1% penicillin/streptomycin (P/S) and 10% fœtal bovine serum (FBS) for HEK293T, HEK293, MDA-MB-231, and HeLa cells. MCF7 were cultured DMEM F-12 medium, 1% P/S and 10% FBS supplemented with 10 μg/mL human recombinant insulin and 0.5 nM estradiol. MCF10A were cultured DMEM F-12 medium containing 1% P/S, 5% horse serum heat inactivated, 10μg/mL Epidermal Growth Factor (EGF), 1 μM Dexamethasone, and 5μg/mL human recombinant insulin. THP-1 and Raji cells were cultured in RPM1 1640 1X (+L-Glutamine) supplemented with 0.05mM 2-mercaptoethanol and 10% fœtal bovine serum. MRC-5 cells were grown in Eagle’s Minimum Essential Medium (MEM; Sigma) supplemented with 10% FBS, 1% P/S, 1X amino acids and 1mM sodium pyruvate.

### Method details

#### Cell transfection

Transfections of HEK293T cells were performed using Lipofectamine 3000 when the cells were 70–80% confluent. Eight hours post-transfection, the medium was replaced with normal growth medium, and lysates were usually prepared at 24h post-transfection.

#### DNA cloning

Clones were prepared in a pcDNA3 backbone. All mutations and deletions were introduced by PCR. The oligos employed are listed.

#### Polysome gradient/RNA extraction

For polysome profiling, 20–60% sucrose (Sigma) in 100 mM KCl, 5 mM MgCl_2_, 20 mM HEPES and 2 mM DTT gradients were prepared manually in SW41 rotor tubes. Cells were treated for 5 min with 50 μg/mL cycloheximide and then collected in cold PBS containing 100 μg/mL cycloheximide. Cells were pelleted and lysed in polysome lysis buffer (100 mM KCl, 50 mM Tris–HCl pH 7.6, 1.5 mM MgCl_2_, 1 mM DTT, 1 mg/mL Heparin, 1.5% (v/v) NP-40, 100 μM cycloheximide, 1% aprotinin, 1 mM AEBSF and 100U/mL of RNasin) supplemented with protease cocktail inhibitor EDTA-free, on ice for 20 min. Lysates were cleared by centrifugation (14,000xg) and the supernatants loaded onto the gradients. These were centrifuged for three and a half hours at 35,000 rpm and 4°C. They were collected through an UV-lamp and an Absorbance detector. One mL fractions were recovered using a Foxi Junior Fraction Collector (Isco). RNA was isolated from each fraction by adding an equal volume of TriZol. Samples were mixed and incubated on ice for 15 minutes before addition of 0.3 volumes of chloroform. After centrifugation, the upper phase was collected, and the RNA precipitated with 0.7 volumes of isopropanol. The RNA pellet was resuspended in water.

#### RT-PCR

The RT-PCR was performed using 250 ngs of total or polysomal RNA that were reverse transcribed using 50 U of Superscript II in a total volume of 25 μL at 42 °C for 1 h. Relative mRNA levels were evaluated by semi-quantitative PCR using the Pfu polymerase as detailed previously.^55^ The number of amplifications cycles was first optimised for each primer set and corresponded to the exponential phase.

#### Tricine gel

Gels were prepared as follows: Separating gel: 16% Acrylamide (Acrylamide/bis-acrylamide 19:1), Gel buffer (1M Tris pH 8.45, 0.1% SDS), 8.75%(v/v) Glycerol, 0.04% ammonium persulphate (APS), and 0.05% TEMED.

Stacking gel: 4.2% Acrylamide (Acrylamide/bis-acrylamide 19:1), Gel buffer (0.75M Tris pH 8.45, 0.1% SDS), 0.2% APS, and 0.2% TEMED.

Migration was performed in the following running buffer: 100mM Tris base, 100mM Tricine, 0.1% SDS at pH8.3.

#### Immunoblot

Cytoplasmic extracts were prepared in either hypotonic lysis buffer (20mM Hepes, 5mM MgCl_2_, 1mM DTT, 2mM ATP, protease inhibitor) or CSH buffer (50 mM Tris-Cl pH 7.5, 250 mM NaCl, 1 mM EDTA, 0.1% (v/v) Triton X-100). Whole cell extracts were prepared by resuspending the cell pellet in either X2 sample buffer (125 mM Tris-HCl pH 6.8, 20% (v/v) glycerol, 10% (v/v) β-mercaptoethanol, 5% SDS, 0.025% (w/v) bromophenol blue) for SDS-PAGE, or X2 sample buffer Novex (450mM Tris-HCl pH 8.45, 12% (v/v) glycerol, 4% SDS, 0.00075% Coomassie Blue G, 10% β-mercaptoethanol) for the Tricine gels.

Protein concentrations were determined by Bradford. Twenty μg of protein was resolved on polyacrylamide-SDS gels and electro-transferred to PVDF membranes.

Antibodies used in this study are listed in the accompanying key resources table. Immunoblots were developed using the WesternBright™ Quantum and quantitated using Image Lab. Rabbit polyclonal Abs against the SEP53BP1 protein were generated by ProteoGenix (France) using two peptides that spanned most of the smORF (Figure 3A). Antibodies were affinity purified using the immobilised antigens.

#### Preparation of hypotonic cell extracts

Extracts were prepared following the protocol outlined in Terenin and coworkers.^123^ Actively dividing cells (≈70% confluence) from a 100mm petri dish were scrapped into ice-cold PBS (final volume 1 mL) and recovered by pelleting at 1,000g for 5minat 4°C. They were washed a second time with PBS before resuspending in 200 μL of Lysolecitin lysis buffer (20 mM HEPES-KOH pH 7.4, 100 mM KOAc, 20 mM DTT, 0.1 mg/mL (w/v) lysolecitin) for precisely 1 minute on ice before centrifuging at 10,000g for 10sat 4°C. The pellet was resuspended in an equal volume of ice-cold hypotonic extraction buffer (20 mM HEPES-KOH pH 7.5, 10 mM KOAc, 1 mM MgAc, 4 mM DTT containing complete protease inhibitor cocktail-EDTA minus). Cells were disrupted in a pre-cooled Dounce homogeniser using 20–25 strokes, transferred to an Eppendorf tube, and spun at 10,000g for 10 min at 4°C.

Glycerol gradients were prepared as previously described.^124^ Hyoptonic cell extracts were supplemented with 2mM ATP to ensure proteasome integrity. Extracts were loaded onto a 10–40% linear glycerol gradient in 100 mM KCl, 5 mM MgCl_2_, and 20 mM HEPES pH 7.4 prepared in an SW60 tube. Gradients were centrifuged for 16hat 30,000 rpm in a SW60 rotor at room temperature. After centrifugation, 10 X 400 μL fractions were collected from the bottom of the tube. Proteins were recovered by methanol/chloroform precipitation, resuspended directly in 40μL of X2 sample buffer and analysed by immunoblotting.

#### *In-vitro* transcription and translation

Plasmids were linearized and RNA *in vitro* transcribed using the mMessage mMachine™ T7 Transcription Kit and purified by LiCl precipitation according to the manufacturer’s instructions. Transcripts were capped using the vaccinia capping system according to the suppliers’ protocol and polyadenylated using the polyA tailing kit. The mRNA quality was controlled on a 2% agarose gel. RNA (100 ng) was translated either in a 50% extract wheat germ extract (WGE) or a HEK293T hypotonic cell extract. For WGE, RNAs were translated for 90minat 25°C in a final volume of 20 μL. For HEK293T hypotonic cell extracts, RNAs (100 ng) were translated for 2 hrsat 30°C in a final volume of 10μL. The following translation energy mix buffer was added (10X: 150mM HEPES, 80mM creatine phosphate, 200μg/mL creatine kinase, 10mM ATP, 10mM GTP, 200μM amino acid mix, 5mM spermidine, 200μg/mL calf liver tRNA, 10mM MgOAc, 10mM DTT). The reactions were stopped by the addition of Laemmli SB buffer (X4 buffer: 250 mM Tris HCl pH 6.8, 40% glycerol, 20% β-mercaptoethanol, 10% SDS, 0.05% Bromophenol blue.

#### Indirect immunofluorescence using confocal imaging and z-stacking

SEP53BP1 was transiently expressed in HEK293T cells grown on No. 1.5 coverslips. Cell suspensions of THP-1 and Raji were pelleted and resuspended in PBS. They were allowed to adhere on the coverslips by sedimentation for 30 minutes at RT. Cells were fixed with 4% (v/v) paraformaldehyde (PFA), permeabilised with 0.3% (v/v) Triton/PBS and then incubated in blocking buffer (2% w/v BSA/PBS) for 60 minutes at RT. Blocking buffer was replaced by the SEP53BP1 primary antibody (1:100 dilution in blocking buffer) overnight at 4°C. Cells were washed X3 with PBS and incubated in the dark for 60 minutes at RT with Alexa Fluor 594 goat anti-rabbit IgG (H+L) (1:500 dilution in blocking buffer). Coverslips were washed X3 with PBS. Nuclei were stained with DAPI (1:1000 in PBS) at RT for 10 minutes in the dark. Coverslips were mounted using Prolong Diamond Antifade Mountant and sealed with nail polish.

Confocal images were collected on a Zeiss LSM800 confocal scanning microscope equipped with a Plan-Apochromat 63x/1.40 Oil DIC M27 objective. Pictures were analysed using the ImageJ software. Z-seriesvideos in THP1 and RAJI cells are shown as maximum z-projections, and gamma, brightness, and contrast were adjusted (identically for compared image sets) and were generated using the Imaris software.

#### siRNA mediated KD

MCF7 cells (∼3x10^7^:50–60% confluence) were transfected with either a scrambled siRNA or a siRNA targeting the *TP53BP1* gene using the RNAiMAX reagent. The sequences of the siRNA molecules are indicated in the key resources table. The transfection protocol was as described previously.^75^ Cells were harvested at 48 h post-transfection by scraping into CSH buffer.

#### Yeast-two-hybrid (Y2H)

Y2H was performed by Hybrigenics Services (https://www.hybrigenics-services.com/) against the Human B cell Lymphoma library.

#### Co-immunoprecipitation (co-IP)

##### α4pull-down

SEP53BP1 was transiently expressed in HEK293T cells. Cells were lysed in hypotonic lysis buffer (20 mM HEPES-KOH pH 7.5, 10 mM KOAc, 1 mM MgAc, 4 mM DTT containing complete protease inhibitor cocktail-EDTA minus) containing 2mM ATP. 500 μg of protein were pre-incubated with α4 antibody overnight at 4°C. The next day, the lysates were incubated with 10 μL of Dynabeads Protein G for 1hat 4°C. Beads were washed X3 in hypotonic lysis buffer, resuspended in X2 sample buffer and analysed by SDS-PAGE.

##### UBQLN4 pull-down

HEK293T cells were co-transfected with pcDNA3 clones expressing SEP53BP1 and UBQLN4. They were lysed in hypotonic lysis buffer. 500 μg of protein were pre-incubated with UBQLN4 antibody overnight at 4°C. The next day, the lysates were incubated with 10 μL of Dynabeads Protein G 1hat 4°C. Beads were washed X3 in hypotonic lysis buffer, resuspended in X2 sample buffer and analysed by SDS-PAGE.

#### Co-immunoprecipitation coupled to mass spectrometry (CoIP-MS)

HEK293T cells seeded on 3 × 10 cm petri dishes were transfected with either a pcDNA expressing SEP53BP1-3HA or the empty vector. At 24 h post-transfection, cells were lysed in 0.5 mL/dish of CSH buffer. The pooled extracts (1.5 mLs containing ∼15 mg of protein) were incubated ON with 30 μL of anti-HA magnetic beads. Beads were washed x3 CSH and x2 with 50 mM NH_4_OAc. Biological duplicates were prepared at one-week intervals. The immobilisation of SEP53BP1-3HA to the beads was confirmed by immunoblotting using 1/50th of the final wash bead suspension. The beads were stored at 0°C before sending to the Functional Genomics Centre Zurich (FGCZ) for LC-MS analysis.

### Quantification and statistical analysis

The LC-MS data was processed using the FragPipe proteomics pipeline. The protein quantification results were extracted from the combined_protein.tsv file (Table S1). In order to score potential interactions between observed proteins (potential prays) and the bait protein the SaintExpress software was employed. The Bait Prey interaction candidate lists were filtered using a Bayesian false discovery rate (BFDR) threshold of 0.05 and an empirical fold-change score (EFC) threshold of 2 (Table S2).

Transcriptome data was extracted from the ENCODE site (https://www.encodeproject.org) selecting only human datasets that had undergone validation and initial filtering. Mean and standard deviations were calculated directly on EXCEL. The numbers of samples are indicated in the figure legend.

## Data Availability

• The raw and processed co-IP mass spec data are in the excel files included in the manuscript (Tables S1 and S2). Microscopy data reported in this paper will be shared by the lead contact upon request. • This paper does not report original code. • Any additional information required to reanalyse the data reported in this paper is available from the lead contact upon request.

## Supplementalinformation

Document S1. Figures S1–S6

Table S1. Excel file with the LC-MS data processed using the FragPipe proteomics pipeline, related to Figure 8

Table S2. Excel file with LC-MS filtered Bait Prey candidate interaction list, related to Figure 8
