# Supplementary material for: TP53BP1, a dual-coding gene, uses promoter switching and translational reinitiation to express a smORF protein
Source: iScience. 2023 Apr 27;26(5):106757. doi: 10.1016/j.isci.2023.106757 (PMC10193022; doi:10.1016/j.isci.2023.106757)
Supplement: Document S1. Figures S1–S6 [file mmc1.pdf]

iScience, Volume *26*

Supplemental information

TP53BP1, a dual-coding gene, uses promoter switching and translational reinitiation to express a smORF protein

Marta A. Inchingolo, Aurélie Diman, Maxime Adamczewski, Tom Humphreys, Pascale Jaquier-Gubler, and Joseph A. Curran

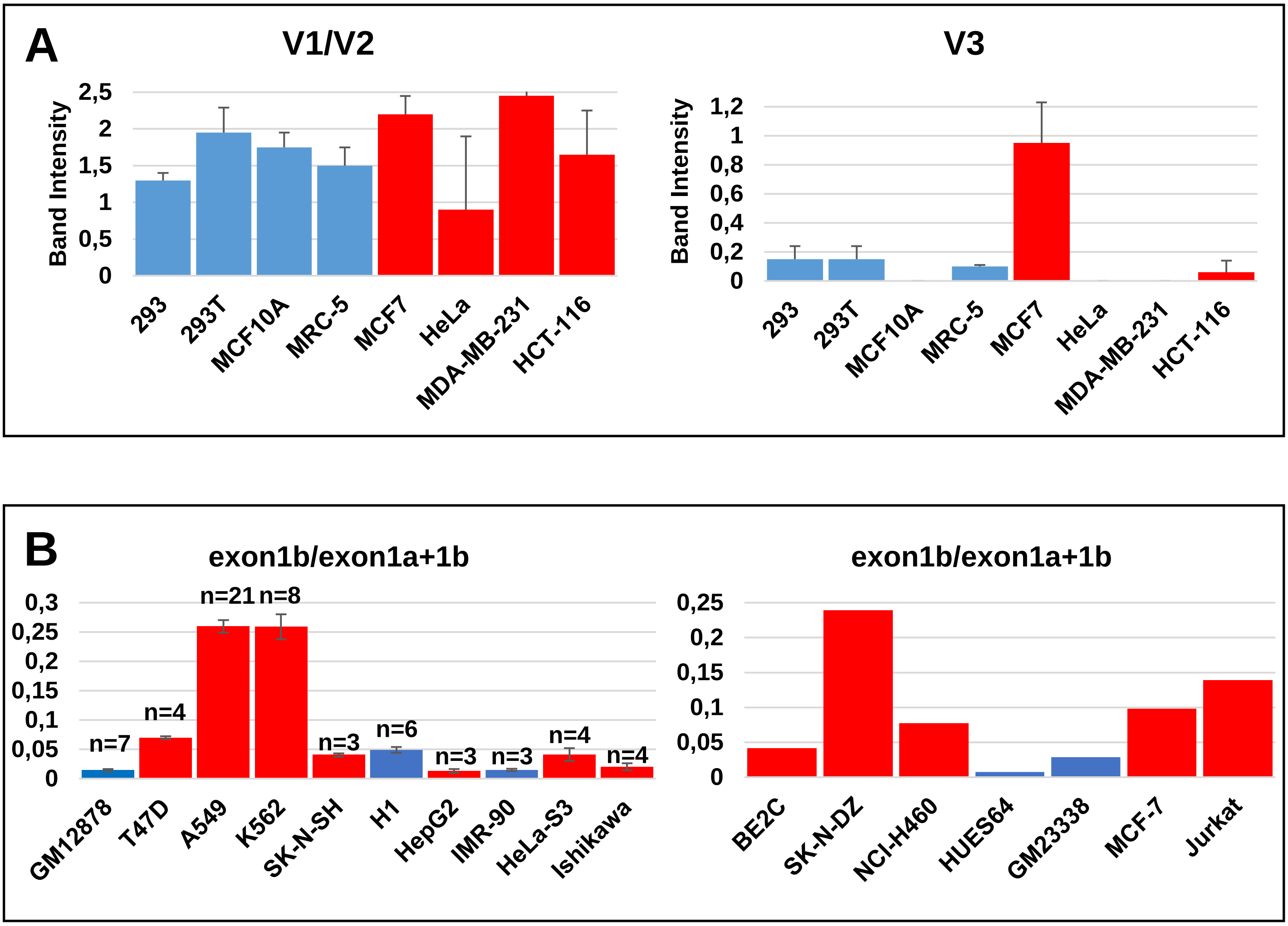

A
V1/V2
Band Intensity
2,5
2
1,5
1
0,5
0
293
293T
MCF10A
MRC-5
MCF7
HeLa
MDA-MB-231
HCT-116
V3
Band Intensity
1,2
1
0,8
0,6
0,4
0,2
0
293
293T
MCF10A
MRC-5
MCF7
HeLa
MDA-MB-231
HCT-116
B
exon1b/exon1a+1b
0,3
0,25
0,2
0,15
0,1
0,05
0
n=7
n=4
n=21
n=8
n=3
n=6
n=3
n=3
n=4
n=4
GM12878
T47D
A549
K562
SK-N-SH
H1
HepG2
IMR-90
HeLa-S3
Ishikawa
exon1b/exon1a+1b
0,25
0,2
0,15
0,1
0,05
0
BE2C
SK-N-DZ
NCI-H460
HUES64
GM23338
MCF-7
Jurkat

Figure S1 The human 53BP1 variants and their expression levels in tumoural and non-tumoural cell lines, related to Figure 1.

(A) Levels of each transcript variant in a range of cell lines available in the lab. RT-PCR was performed with primer sets specific for the V1/2 and V3 variants. Amplicons were analysed on agarose gels and band intensities (arbitrary units) were normalised to an actin amplicon control. The results are from biological triplicates. Bars indicate the SD.

(B) The abundance of the exon 1b (derived from the P2 promoter and present in V3: exon 1a arises from the P1 promoter and is present on V1/2) relative to total exon 1 expression (P1 + P2 or exon 1a + 1b) from a series of cell lines. Transcriptome data was extracted from the ENCODE site (https://www.encodeproject.org) selecting only data sets that had undergone validation and initial filtering. The total number of reads corresponding to each exon 1 was determined and used to estimate the fraction arising from the P2 promoter (reads exon 1b/reads 1a+1b). The left hand panel shows cell lines in which multiple independent data sets were available (indicated by n above the columns). SD values are shown. The right hand panel shows cell lines for which only a single study was available. In both panels red columns indicate tumoural and blue columns non-tumoural cells.

53BP1 (N-term)

```
HUMAN       MDPTGSQLDSDFSQQDTPCLIIEDSQPESQVLEDDSGSHFSMLSRHLPNLQTHKENPVL--DVVSNPEQTAGEERGDGNSGFNEHLKENKVA-DPVDSSNLDTCGSISQVIEQLPQPN
Chimp       MDPTGSQLDSDFSQQDTPCLIIEDSQPESQVLEDDSGSHFSMLSRHLPNLQTLKENPVL--DVVSNPEQTAGEERGDGNSGINEHLKENKVA-EPVDSSNLDTCGSISQVIEQLPQPN
Rattus      MDPTGSQLDSDFSQQDTPCLIIEDSQPESQALEEDAGSHFSVLSRHLPNLQMHKENPVL--DIMSNPEQTVA-EQGDN-SSFSEHLKEKKAS-DPVESSHLGTSGPISQVIERLPQPN
Green       MDPTGSQLDSDFSQQDTPCLIIEDSQPESQVLEDDSGSHFSMLSRHLPNLQTHKENPVL--DVVSNPEQTAGEERGDSNSGFNEHLKENKVA-DPVDSSNLDTCGSVSQVIEQLPQPN
Orangutan   MDPTGSQLDSDFSQQDTPCLIIEDSQPESQVLEDDSGSHFSMLSRHLPNLQTHKENPVL--DVVSNPEQTAGEERGDSNSGFNEHLKENKVA-DPMDSSNLDTCGSISQVIEQLPQPN
Squirrel    MDPTGSQLDSDFSQQDTPCLIIEDSQPESQVLEDDSGSHFSMLSRHLPNLQTHKENPVL--DVVSNPEQTAEEERGDSNREFNEHLKENKVA-DPVDSSNLDTCGSISQVIEQLPQPN
Snub        MDPTGSQLDSDFSQQDTPCLIIEDSQPESQVLEDDSGSHFSMLSRHLPNLQTHKENTVL--DVVSNPEQTAGEERGDSNSGFNEHLKENKVA-DPVDSSNLDTCGSISQVIEQLPQPN
Macaque     MDPTGSQLDSDFSQQDTPCLIIEDSQPESQVLEDDSGSHFSMLSRHLPNLQTHKENPVL--DVVSNPEQTAGEERGDSNSGFNEHLKENKVA-DPVDSSNLDTCGSISQVIEQLPQPN
Sooty       MDPTGSQLDSDFSQQDTPCLIIEDSQPESQVLEDDSGSHFSMLSRHLPNLQTHKENPVLVTDVFKKPKTTTGEERGDSNSGFNEHLKENKVA-DPVDSSNLDTCGSISQVIEQLPQPN
Tarsier     MDPTGSQLDSDFSQQDTPCLIIEDSQPESQVLEDDSGSHFGVLSRHLLNLQTHKENPVL--DVVSNPERTAGEEQGDSNSGFSEHLKENKAA-DAIDSSHMDACGSISQVIEQLPQPN
Tarsius     MDPTGSQLDSDFSQQDTPCLIIEDSQPESQVLEDDSGSHFGVLSRHLLNLQTHKENPVL--DVVSNPERTAGEEQGDSNSGFSEHLKENKAA-DAIDSSHMDACGSISQVIEQLPQPN
Mouse       MDPTGSQLDSDFSQQDTPCLIIEDSQPESQVLEDDSGSHFGVLSRHLPNLQTHKENPVL--DVVSNPEQTAGEEQGDSNSGFSEHLKENKAA-DSMDSSHLDTCGSISQVIEQLPQPN
Squirrel    MDPTGSQLDSDFSQQDTPCLIIEDSQPESQVLEDDSGSHFSMLSRHLPNLQTHKENPVL--DVVSDPEQAAGEEQGNSNSGFTEHLKD-KAA-VPVDSSHLDTCGSISQVIEQLPQPN
Hamster     MDPTGSQLDSDFSQQDTPCLIIEDSQPESQVLEEDAGSHFSVLSRHLPNLQIHKENPVL--DIVSNPDQAAAEEQGDNNSSF-KHLKENKASADPVDSSHLGTCDSISQIIEQLPQPN
Rat         MDPTGSQLDSDFSQQDTPCLIIEDSQPESQALEEDAGSHFSVLSRHLPNLQMHKENPVL--DIMSNPEQTVA-EQGDSNSSFSEHLKEKKAS-DPVESSHLGTSGPISQVIERLPQPN
Mole        MDPTGSQLDSDFSQQDTPCLIIEDSQPESQVLEDDSGSHFSVLSRHLPNLQLHRENPVL--DAVSNPEQTT--EQGDRNSGFSEHLKENKAA-DPMDSSHLDTCSSISQVIDRLPQPN
Pig         MDPTGSQLDSDFSQQDTPCLIIEDSQPESQVLEDDSGSHFSMLSRHLPNLQTHKENPVL--DVVSNTEQTGGEEQGDDNSSFNECLKENKAA-DSMDSSHLDTCGSISQVIEQLPQPN
Elephant    MDPTGSQLDSDFSQQDTPCLIIEDSQPESQVLDD-SGSHFSMLSRHLPNLQTHKENPVL--DVVSNPEQAAGEEQGDNNSGSNEHLKENKPS-EPMDSSHLDTCSSVSQVIEQLPQPN
Whale       MDPTGSQLESDFSQQDTPCLIIEDSQPESQVLDDDSGSRFSMLSRHLPNLQTHKENPLL--DVVSNSEQTAGEEQGDSNSGFNEQLKENKAA-ESMDSSHLDTCGSISQVIEQLPQPN
Cat         MDPTGSQLDSDFSQQDTPCLIIEDSQPESQVLEDDSGSRFSLLSRHLPNLQTHKENPVL--DVVSNPEQTAGE-GGDSNSGFNEDLKENKAA-DPMESSHLDTGGTISQVIEQLPQPN
```

SEP53BP1

```
HUMAN               MILVLTSVCYLDTFLISRRTKKILCWMLCPILNKQLEKNEETVIVGSMNI
Chimp               MILVLTSVCYLDTFLISRRSKKILCWMLCPILNKQLEKNEETVIVGSMNI
Green_Monkey        MILVLTSVCYLDTFLIFRRTKKILYWMLCPILNKQLEKNEETVIVGSMNI
Orangutan           MILVLTSVCYLDTFLISRRTKKILCWMLCPTLNKQLEKNEETVIVGSMNI
Squirrel_Monkey     MILVLTSVCCLDTFLIFRRTKKILCWMLCPILNRQLKKNEETVIGSSMNI
Snub-nosed_monkey   MILVLTSVCYLDTFLIFRRTKKIPYWMLCPILNKQLEKNEETVIVGSMNI
Macaque             MILVLTSVCYLDTFLIFRRTKKILYWMLCPILNKQLEKNEETVIVGSMNI
Sooty_mangabey      MILVLTSVCYLDTFLIFRRTKKILYWMLCPILNKQLEKNEETVIVGSMNI
Tarsier             MILGLTSVCSLDTFLIYRRTKRTLCWMLCPILNEQLEKNKETVIVGSVNI
Tarsius_syrichta    MILGLTSVCSLDTFLIYRRTKRTLCWMLCPILNEQLEKNKETVIVGSVNI
Mouse_Lemur         MILVLTLVCCLDTFLTYRRTKRILCWMLCPILNKQLEKNREIVIVGSVNI
Squirrel            MILVLTSVCCLDTFRISRRTKRTLCWMLCPILNKLLEKNKKTVIVGSVNI
Hamster             -MRALTSVCCLDTFPICRYTKRTLCWILCPIRNKQLQKSKETIIAPSMNI
```

Figure S2 ClustalW2 Alignments used to generate data related to Figure 2A.

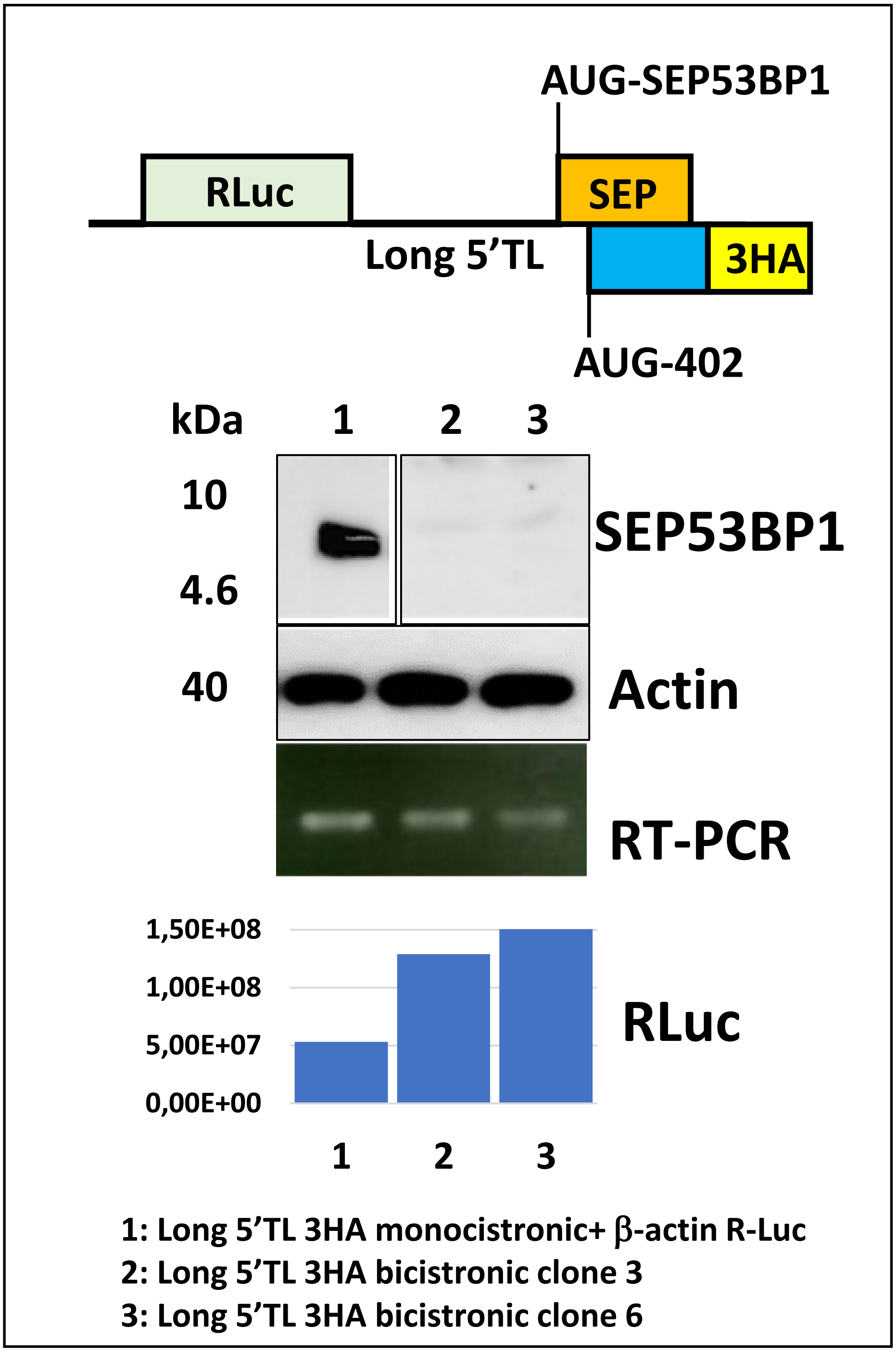
AUG-SEP53BP1
RLuc
SEP
Long 5'TL
3HA
AUG-402
kDa
1
2
3
10
4.6
SEP53BP1
40
Actin
RT-PCR
1,50E+08
1,00E+08
5,00E+07
0,00E+00
RLuc
1
2
3
1: Long 5'TL 3HA monocistronic+ β-actin R-Luc
2: Long 5'TL 3HA bicistronic clone 3
3: Long 5'TL 3HA bicistronic clone 6

Figure S3 The human Long 5'TL does not drive second cistron expression in a plasmid-based bicistronic assay, related to Figure 4.

We generated a bicistronic construct with a first cistron renilla (Rluc) fused upstream of the Long 5'TL-3HA sequence, as depicted in the upper panel. Independent clones of this construct were transiently expressed in HEK293T. In parallel cells were transfected with the monocistronic Long 5'TL-3HA plus a monocistronic β-actin R-Luc. SEP53BP1 and actin levels were monitored by immunoblotting and transcript levels by RT-PCR using a primer set specific for the SEP53BP1 region. Renilla activity in each extract was also recorded. All transcripts carried 5'TL sequences derived from β-actin. Lanes 1, and 2/3 are separates lanes from the same gel.

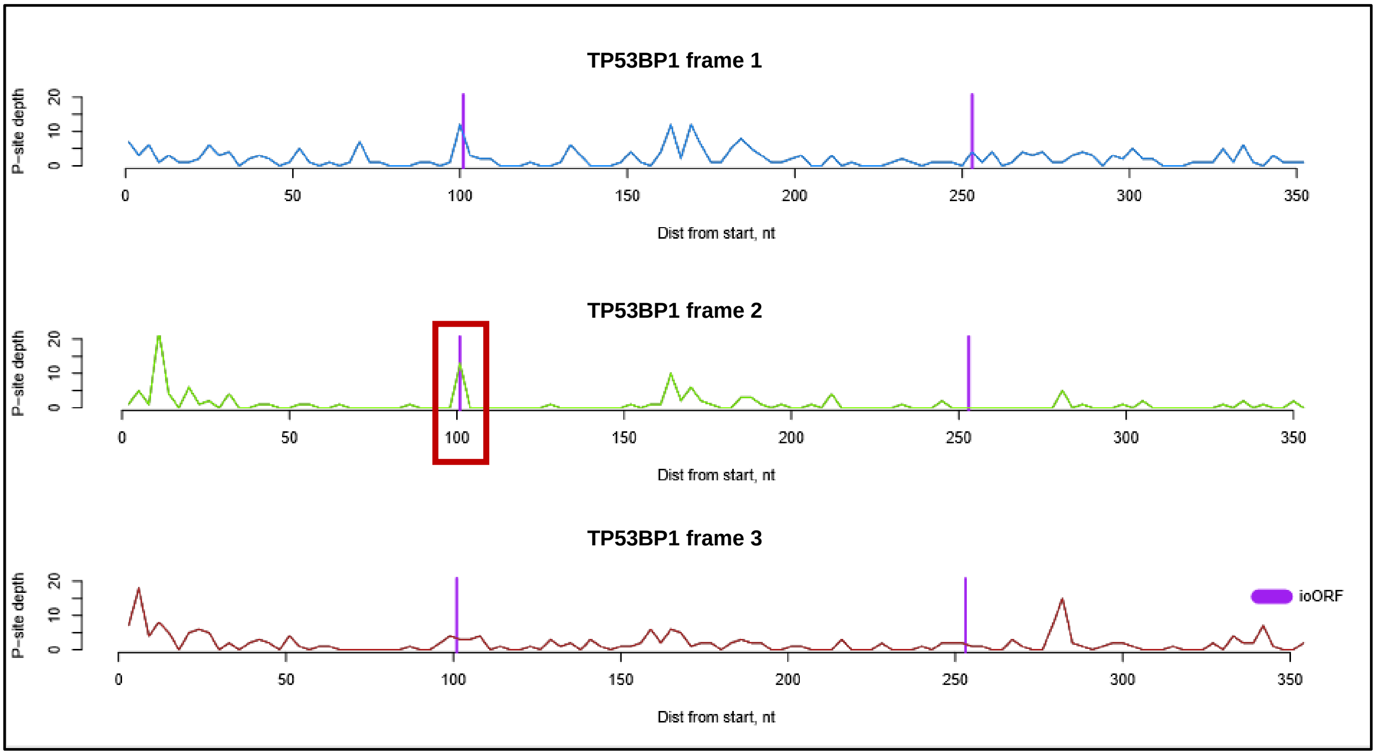

TP53BP1 frame 1
P-site depth
0
10
20
0
50
100
150
200
250
300
350
Dist from start, nt
TP53BP1 frame 2
P-site depth
0
10
20
0
50
100
150
200
250
300
350
Dist from start, nt
TP53BP1 frame 3
P-site depth
0
10
20
ioORF
0
50
100
150
200
250
300
350
Dist from start, nt

Figure S4 Ribosome Profiling across the SEP53BP1 ioORF region in THP-1 cells. (the violet vertical lines indicate the AUG start and UGA stop sites of the ioORF: the +1 position starts after the $AUG^{53BP1(b)}$), related to Figure 5.

The data was extracted from the RPF database http://sysbio.sysu.edu.cn/rpfdb/index.html. The read scoring follows P-site occupancy across the three possible ORFs, with frame 1 corresponding to that of 53BP1 and frame 2 to that of SEP^{53BP1}. The red rectangle highlights the increase in P-site read count that places the AUGSEP53BP1 start codon in the P site.

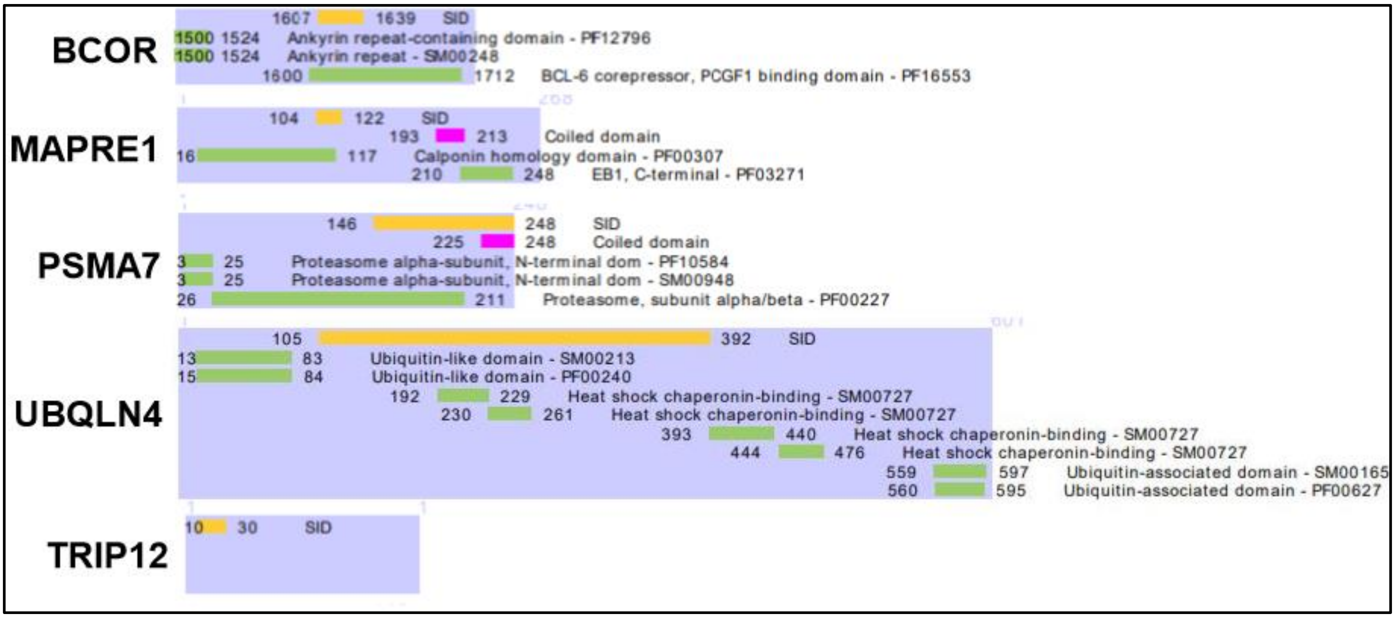
BCOR
1607 1639 SID
1500 1524 Ankyrin repeat-containing domain - PF12796
1500 1524 Ankyrin repeat - SM00248
1600 1712 BCL-6 corepressor, PCGF1 binding domain - PF16553
MAPRE1
104 122 SID
193 213 Coiled domain
16 117 Calponin homology domain - PF00307
210 248 EB1, C-terminal - PF03271
PSMA7
146 248 SID
225 248 Coiled domain
3 25 Proteasome alpha-subunit, N-terminal dom - PF10584
3 25 Proteasome alpha-subunit, N-terminal dom - SM00948
26 211 Proteasome, subunit alpha/beta - PF00227
UBQLN4
105 392 SID
13 83 Ubiquitin-like domain - SM00213
15 84 Ubiquitin-like domain - PF00240
192 229 Heat shock chaperonin-binding - SM00727
230 261 Heat shock chaperonin-binding - SM00727
393 440 Heat shock chaperonin-binding - SM00727
444 476 Heat shock chaperonin-binding - SM00727
559 597 Ubiquitin-associated domain - SM00165
560 595 Ubiquitin-associated domain - PF00627
TRIP12
10 30 SID

Figure S5 The SEP53BP1 partners as determined by Y2H are represented according to their protein chain length, related to Figure 7.

The selected interaction domains (SID) and the known functional domains of each protein are all indicated.

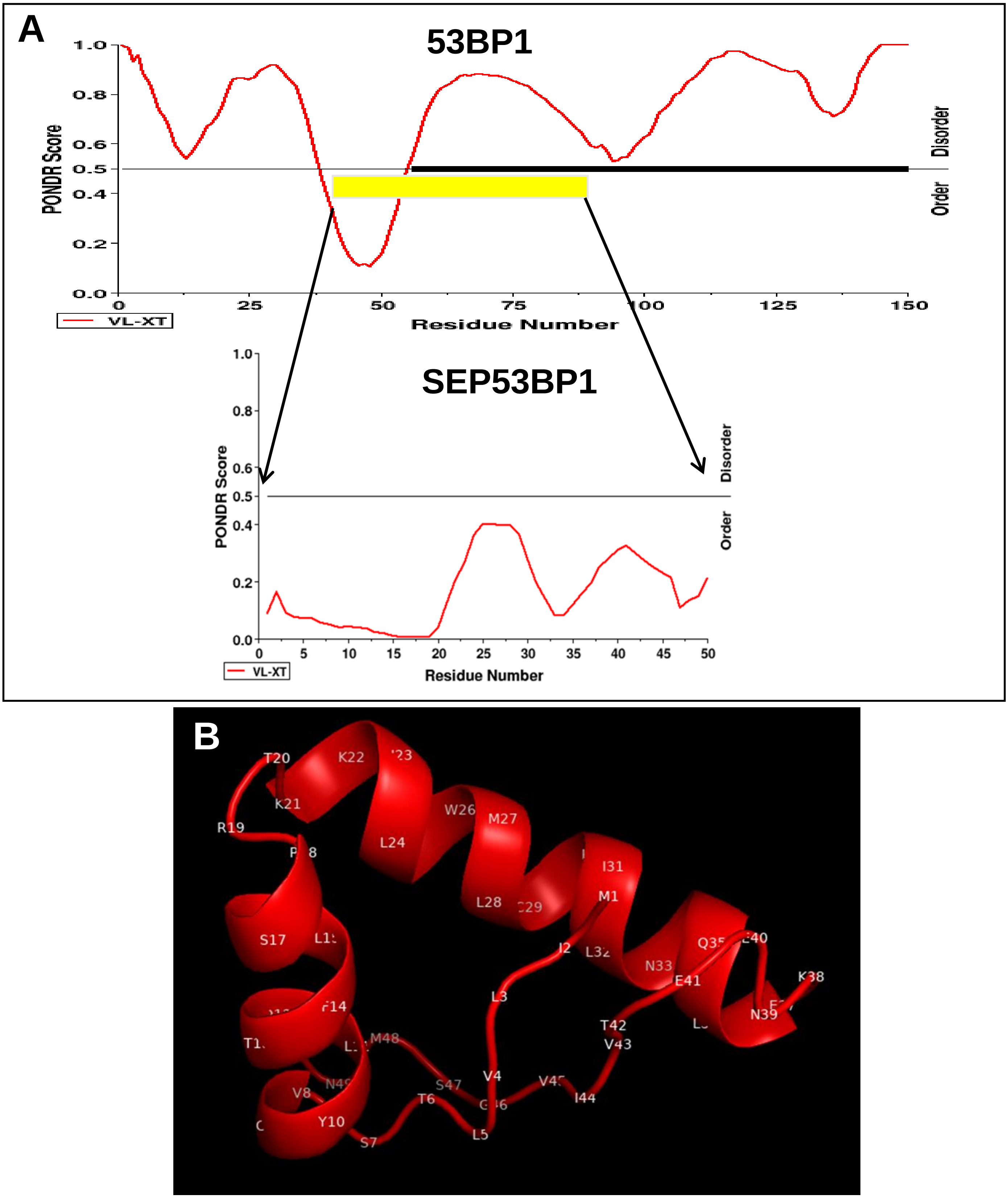
A
53BP1
PONDR Score
1.0
0.8
0.6
0.5
0.4
0.2
0.0
0
25
50
75
100
125
150
Residue Number
VL-XT
Disorder
Order
SEP53BP1
PONDR Score
1.0
0.8
0.6
0.5
0.4
0.2
0.0
0
5
10
15
20
25
30
35
40
45
50
Residue Number
VL-XT
Disorder
Order
B
T20
K22
K21
R19
L24
W26
M27
I31
L28
M1
S17
I2
L32
N33
E41
K38
L3
N39
T42
V43
M48
V4
S47
T6
V8
Y10
S7
L5
I44
E40
F14

Figure S6 Structural predictions for the human SEP53BP1 protein, related to Figure 6.

(A) Structural disorder as measured by PONDR (Predictor of Natural Disordered Region) score (http://www.pondr.com). The upper panel covers the N-terminal 150 aa of 53BP1 (starting from the AUG^{b} start codon) with the positioning of the overlapping SEP53BP1 ORF indicated in yellow. The disorder prediction for the SEP ORF is indicated below.

(B) A three dimensional tertiary structural model generated using I-TASSER (Iterative Threading ASSEmbly Refinement: https://zhanggroup.org).
